# Enhancement of commercial dye photocatalytic degradation through the use of silver-doped kaolinite-zinc oxide quantum dots

**DOI:** 10.1186/s13065-025-01648-2

**Published:** 2025-10-29

**Authors:** Amr M. Farrag, Mahmoud E. Awad, Mohamed M. Aboelnga, Ashraf A. El-Bindary

**Affiliations:** 1 Chemistry Department, Faculty of Science, Damietta University, New Damietta, 34517 Egypt; 2 Applied Mineralogy Lab, Geology Department, Faculty of Science, Al-Azhar University, Nasr City, Cairo, 11884 Egypt; 3 Chemistry Department, Faculty of Basic Sciences, King Salman International University, South Sinai, Ras Sudr, 46612 Egypt

**Keywords:** Photocatalytic degradation, Quantum dots, Scavenger, Box-Behnken design

## Abstract

To manufacture (Ag)Kao/ZnO-QDs, which are utilized to remove rhodamine B (RhB) and indigo carmine (IC) dyes, we present a simple pyrofabrication method for silver-doped kaolinite-zinc oxide quantum dots. A homogenous combination between zinc nitrate hexahydrate, silver nitrate besides kaolinite was completely mixed then heated gradually till 400 °C to create the very effective photocatalyst known as (Ag)Kao/ZnO-QDs. TEM, SEM–EDX, XRD, FTIR, XPS, ZP, UV–Vis DRS and BET were among the examination techniques used to analyze the **(**Ag)Kao/ZnO-QDs nanoparticle. To investigate the effectiveness of the (Ag)Kao/ZnO-QDs photocatalyst for elimination of IC and RhB dyes in aqueous solutions, various pH dye solutions were exposed to solar light during ambient conditions. A total pore volume of 0.0769 cc/g, an enhanced particular surface area of 50.3148 m^2^g^− 1^ and a pore size of 3.0565 nm were all characteristics of the generated nanostructure. Furthermore, 2.89 eV was found to be the optical band gap energy. Following 150 min of exposition to solar irradiation, the study indicates that the (Ag)Kao/ZnO-QDs nanostructure with optimal dose of 2000 and 2500 mg/L achieved the highest removal performance of IC and RhB dyes at 93.92 and 96.93%, respectively, at neutral conditions (pH = 7). The dye removal process of IC and RhB dyes followed first-order kinetics with rate constants of 0.0166 and 0.018 min^− 1^ at pH = 7, individually. Scavenger radical tests have demonstrated that superoxide radicals (^•^O_2_^−^) are essential to the mechanism of the decomposition of IC dye. While superoxide radicals (^•^O_2_^−^) plus hydroxyl radicals (^•^OH) are necessary for the photocatalytic degradation pathway of RhB dye. After triple reuse studies, it was showed that the (Ag)Kao/ZnO-QDs nanoparticle was highly stable as evidenced by the photodegradation efficacy of IC and RhB dyes which went from 93.92 to 92.78, 91.54, and 89.76% as well as from 96.93 to 95.08, 94.11 and 92.58%, individually. The Box-Behnken design (BBD) which identified the optimal values for a number of variables was appropriate for the photocatalytic degradation tests.

**Supplementary Information:**

The online version contains supplementary material available at 10.1186/s13065-025-01648-2.

## Introduction

Water that is safe for drinking will become increasingly scarce and costly due to contamination brought on by industry and increasing numbers of people [1]. The most recent World Health Organization (WHO) survey estimates that 844 million persons globally do not have accessibility to clean drinking water [2]. Numerous illnesses such as cholera, hepatitis and typhoid are spread by pathogens carried by water which are illness-causing bacteria or viruses. Because water contamination is not well managed, unclean water contributes to infections in developing nations. The presence of organic contaminants in sewage can be destructive to all kinds of life. People as well as animals that regularly consume polluted or partially filtered water get cancer or suffer from chronic illnesses [3]. Consequently, water recycling and sewage cleanup have become the main areas of scientific interest. In regions filled by dryness, specific improvements could be made to the regulations governing water pollution and the reusing of sewage [4, 5].

Dyes are among the many new substances that could be used in a variety of manufacturing procedures like paper and textile which would lead to significant pollution. According to numerous studies, textile and manufacturing dyes are among the most common families of toxic chemical substances [6]. The World Bank reports that the coloring and finishing of textiles sectors are responsible for about 17–20% of water contamination. Of the primary sewage pollutants which had been detected, it was revealed that 72 compounds were released during the clothing coloring process with about 30 of those substances being incurable [7, 8]. According to numerous studies, the textile industry uses 10–12% of dyes each year including Victoria blue, Rhodamine B, Indigo Red, Rose Bengal, Indigo Caramine, Eriochrome, Red 120, and Methylene Blue. Of these, a significant amount (20%) is missing through manufacturing and usage and finishes in sewage [9, 10]. These dye-contaminated waste products are made up of colorful, extremely toxic and unsustainable pigments which are bad for all life on earth. Even at extremely low quantities, dyes are readily apparent in water and contaminate aquatic habitats [11]. Dyes greatly irritate the skin, eyes and respiratory system and drinking water polluted with dyes is extremely carcinogenic. Among the major adverse effects of dyes are difficulty in breathing, swelling of face, lips, tongue, mild rise in blood pressure and sometimes bronchoconstriction can happen. Because they exhibit carcinogenic characteristics that can result in acute damage to reproductive, developmental and neurological organs. Further, it has been linked to the disturbance of major neurological systems as well as various critical organs, such as the kidney, liver and brain [12, 13]. Additionally, A significant number of synthetic dyes are discharged into the environment throughout the textile platform dyeing process since they have no attraction for the surfaces that need to be dyed. Textile manufacturing effluents are highly salinized and toxic, especially when it comes to waste gasses released by water worn and contamination of water. In the water and soil of rivers that receive treated wastewater, the dyes may undergo partial degradation or transformation. Furthermore, dyes and the byproducts of their degradation are carcinogenic, teratogenic and mutagenic. Additionally, chlorine that is organically bonded is present in around 40% of dyes worldwide [14, 15]. Therefore, it’s critical to remove pigments from sewage.

In the past few years, eliminating dyes along with various contaminants from water has grown more difficult [16]. Therefore, a number of approaches such as bioadsorption, ozonation, adsorption, ion exchange removal, biological/aerobic treatment, photocatalytic degradation and catalytic reduction had been used to address this specific issue [17, 18]. Each of these processes has advantages or disadvantages in comparison to the other. In order to ascertain how choosing an appropriate method to degrade the dye in issue is efficient, a workable process must be taken. The adsorption procedure is typically preferred when it concerns in dye elimination techniques because it is simple to use and inexpensive. In modern adsorption techniques, common adsorbents such as carbon nanotubes, activated carbon, and raw clay minerals (kaolinite, bentonite, montmorillonite and sepiolite) in addition to their modified forms have been employed [19, 20]. Nevertheless, the inability to remove contaminants completely by this way, lack of adsorption efficiency and the weak stability of adsorbents make it difficult to remove wastes effectively [21, 22].

In recent times, photocatalytic degradation method of contaminants has gotten extremely widespread. The photodegradation procedure includes heterogeneous catalytic processes in which solar irradiation is absorbed by a semiconductor catalyst to break down a variety of external harmful substances such as organic compounds in the air and water. Photodegradation offers benefits over conventional wastewater disposal techniques. For instance, reactive composites can completely degrade organic contaminants at ambient temperature through a little period of time. Furthermore, organic contaminants could entirely breakdown into comparatively not harmful substances (H_2_O and CO_2_) without creating of further harmful compounds [23, 24]. Nanoparticle’s surfaces are functionalized for photodegradation which efficiently breaks down dangerous contaminants. The sole processes needed for the photocatalytic degradation procedure are oxidation and reduction (h^+^ and e^−^) reactions produced on the outside layer of the composites by ultraviolet (UV) or visible irradiation. Wherefore, the ideal nanocomposite materials must be inexpensive, easily accessible as well as environmentally friendly [25, 26].

Several popular clay minerals including kaolinite, chlorite and bentonite are being used to produce inorganic nanocomposites due to their extensive natural existence, human security, little cost and exceptional physical and chemical surface properties [27, 28]. With at least one dimension between 1 and 100 nanometers, nanoparticles provide enhanced performance, reactivity and flexibility because of their nanoscale dimensions. For uses like photocatalysis, adsorption and drug administration that call for significant surface activity, these properties make them perfect choices. Moreover, there are many advantages of nanoparticles compared with other materials including: huge surface area and more active sites which are not present in bulk materials. Because they are very simple to produce, nanoparticles are utilized in drugs to target specific areas. Because of their little dimensions nanoparticles enter tiny capillaries and are absorbed by the cell, enabling effective medication delivery. Adjustable characteristics is one of the most important advantages of nanomaterial which enable scientists to change their shape and morphology. Finally, nanoparticles can fully degrade contaminants without secondary pollutants [29, 30].

Kaolinite is regarded to be among the foremost significant clays utilized in the production of several nanomaterials with a wide range of research and industrial usage. Purified kaolinite is white in coloration with chemical structure consists of 39.50% Al_2_O_3_, 46.54% SiO_2_ and 13.96% H_2_O [31]. Since there is minimal substituent isomorphism in the tetrahedral or octahedral platelets, the chemical structure of kaolinite is [Al_2_Si_2_O_5_(OH)_4_]. The weathering of additional rocks, including feldspars, can release Si and Al, which can lead to the formation of kaolinite in reservoirs. The greatest combination particles (under 2 μm) which comprise kaolinite powders are make up the tiniest layered nanoplatelets (under 200 nm) that have an elasticated layer shape. All kaolinite minerals have the basic 1:1 layered organization which is made up of repeating alumina octahedral and silica tetrahedra plates [32, 33]. Kaolinite can be utilized extensively in many applications now because of its great constancy, large specific surface area and non-toxic nature. Kaolinite is comparatively more acid-tolerant (till pH = 2) and heat-stable (till 400 °C) than various clay minerals. Owing to each the above advantages, researchers are currently interested in using pure kaolinite owing to its easy usage as an affordable and environmentally beneficial photocatalyst in the synthesis of kaolinite-metal oxide material for the elimination of many contaminants [34, 35].

There are several approaches to produce kaolinite-metal oxide and kaolinite-metal nanostructures. Among the most popular fabricating processes, sol-gel, chemical reduction, hydrothermal, co-precipitation and wet impregnation which have been shown to be expensive as well as environmentally unfriendly [36, 37]. The ineffective physical interface among metals and kaolinite due to its weak physical and chemical constancy represents one of the greatest important problems concerning these materials. Furthermore, whenever such nanocomposites are employed as photocatalysts for treating acidic sewage, they result in a considerable amount of metal separation and the resulting poisonousness [38, 39]. Thus, this recent study created a new kaolinite-zinc oxide quantum dots which doped with silver atoms to form (Ag)Kao/ZnO-QDs nanomaterial that is physicochemically steady as well as highly effective. This output has been produced using a quick, inexpensive and environmentally safety pyrofabrication process by combining zinc nitrate hexahydrate (Zn (NO_3_)_2_.6H_2_O) and silver nitrate (AgNO_3_) with a quantity of refined Egyptian kaolinite. The finished nanomaterial has completely described using several types of methods. In order to determine how well the nanocomposite worked as a photocatalyst towards the photodegradation of rhodamine B and indigo carmine dyes under exposure to sunlight, various variables were investigated such as dye concentrations, pHs and photocatalyst quantities.

## Experimental details

### Chemicals

The F1 coded sample showed a high-quality pure kaolin model having manufacturing and commercial value from the Abu Zenima kaolin deposit in Egypt [40]. Sigma-Aldrich provided the employed indigo carmine (C_16_H_8_N_2_Na_2_O_8_S_2_) with pureness of 85%, silver nitrate (AgNO_3_) with pureness ≥ 99%, rhodamine B (C_28_H_31_ClN_2_O_3_) with pureness ≥ 95% and zinc nitrate hexahydrate (Zn (NO_3_)_2_.6H_2_O) with pureness of 98%. Furthermore, sodium hexametaphosphate (Na_6_ (PO_3_)_6_), sodium carbonate (Na_2_CO_3_), iso-propanol ((CH_3_)_2_CHOH), silver nitrate (AgNO_3_), potassium iodide (KI) and P-benzoquinone (C_6_H_4_O_2_) were used and provided from Sigma-Aldrich.

### The Kaolin purification procedure

Prior to the purifying procedure, the initial kaolin sample had been ground into a finer form, dried then screened to reduce its particle diameter to a smaller size below 125 μm. The precipitation procedure was used to purify the tiny raw kaolin powder [41]. An aquatic solution which includes sodium hexametaphosphate (0.75%) in addition sodium carbonate (0.25%) at neutral conditions was mechanically stirred over 180 g of dry kaolin units for 60 min. After that, the suspension was filled into a 500 mL cylinder and left inside for 24 h. Following that, the bright dispersal parts at the highest level were decanted with fineness and high-pureness kaolinite particles. Following the removal of the heaviest minerals impurities from the lowest darker hard section, the bigger kaolinite units remained. This tiny kaolinite combination was cleaned and washed three times further using distilled water then centrifuged at 11,000 rpm. Following the purification procedure, the kaolinite was dehydrated overnight at 75 °C through an electrical furnace and then mashed using a pestle and mortar [42]. Ultimately, the refined kaolinite powder obtained by the earlier process can be used to create the desired (Ag)Kao/ZnO-QDs photocatalyst.

### The process of pyrofabricating the (Ag)Kao/ZnO-QDs structure

Silver doped kaolinite-zinc oxide quantum dots **(**Ag)Kao/ZnO-QDs structure output has been obtained by employing the rapid pyrofabrication technique. The silver nitrate (AgNO_3_) (molar mass = 169.87 g/mol) and zinc nitrate hexahydrate (Zn (NO_3_)_2_.6H_2_O) (molar mass = 297.49 g/mol) that exist in solid forms at room temperature were utilized to determine the catalyst component quantities. Utilizing a ceramic melting-pot, 0.032 g of AgNO_3_ and 0.8 g of Zn (NO_3_)_2_.6H_2_O were completely combined through solid state mixing with 1 g of purified kaolin particles to synthesis (Ag)Kao/ZnO-QDs nanostructure comprising 15% Zn doped with 2% Ag atoms and 83% pure kaolinite. The well combined ingredients were burned in an electrically furnace with regulated temperatures about 38 min in an exposed crucible. The temperature rose with an average of 10 min for each degree Celsius from 20 to 400 °C and it remained at that level for another half hour prior to being permitted to drop down on a systematic basis. Following a mortar crushing, the final nanostructure compound was placed in a plastic bottle to prevent daylight and air. previously the (Ag)Kao/ZnO-QDs photocatalyst product was described and evaluated for the photocatalytic degradation procedure, it was ultimately kept in a black and dryish circumstances [42].

### The (Ag)Kao/ZnO-QDs photocatalyst’s characteristics

The X-ray diffraction (XRD) technique was used to analyze and compare the structural differences among the fabricated (Ag)Kao/ZnO-QDs nanostructure and refined kaolinite sample. The X’Pert Pro diffractometer (CuKα radiation, 45 kV, 40 mA, Malvern PANalytical Ltd, Malvern, Almelo, Netherland) with an X’Celerator solid-state lined detector (Malvern PANalytical Ltd, Malvern, Almelo, Netherland) was used to perform the XRD analysis. The 2θ range covered 5 to 80^o^ via a step growing of 0.008^o^ 2θ and a measurement period of 10 s/step. By using the Scherrer formula, XPOWDER^®^ system had the ability to determine the mineral phases and restrict the mean crystallite size (D001) of both previous models.

XPS spectrum have been applied to investigate the interactions among zinc, silver and kaolinite in the (Ag)Kao/ZnO-QDs nanostructure. The X-ALPHA (Themo Fisher Scientific, USA) was used to create the XPS results using monochromatic X-ray Al K-alpha irradiation − 10 to 1350 eV having a spot diameter of 400 micro mats, pressure of 10^− 9^ mbar, entire spectral crossing power of 200 eV and thin spectrum of 50 eV.

The orientations and structured shifts of the OH groups along the outside edge/basal plane faces of the (Ag)Kao/ZnO-QDs nanocomposite are used to determine the microstructural distribution of embedded nanocrystallized zinc and silver through the kaolinite. Using a SHIMADZU-IRTracer-100 spectrometer and FTIR spectroscopy investigation, this structure feature was successfully detected. A high resolution of 0.25 cm^− 1^ and a fast-scanning rate of 20 spectra per second have been employed for recording spectral observations in the 300–4500 cm^− 1^ wavenumber range. Additionally, conventional EP/CHP/JP/USP/ASTM testing techniques (Japan) are used to evaluate the FTIR functioning [42].

SEM and TEM electron microscopes (JEOL JEM-2100, Japan) operated at 160 kV have been utilized to examine the micromorphological and microtextural images of nanomaterials in the synthesized (Ag)Kao/ZnO-QDs photocatalyst powder model. The sample under investigation was placed on a copper surface. Selective area electron diffraction (SAED) was used to identify the nanostructure of ZnO and Ag_2_O quantum dots. The distribution of elemental zinc and silver was identified through energy-dispersive X-ray spectroscopy (EDX-map) which was outfitted with a 5 kV operation voltage, a SUPER-X silicon-drift window-less EDX detector and a HAADF detector.

Zeta potential analysis can be used to identify the (Ag)Kao/ZnO-QDs photocatalyst’s point of zero surface charge. The zeta potential measurements were determined using the Nanotrac wave II/Q/Zeta equipment (Microtrac MRB, Osaka, Japan). It is based on dynamic light scattering (DLS) using the Frequency Power Spectrum (FPS) distribution technology. 10 mg of dried free kaolinite and (Ag)Kao/ZnO-QDs nanostructure had been then sonicated for ten minutes in 100 mL of deionized water to produce suspensions. By employing 0.1 M HCl and 0.1 M NaOH for changing the pH of both prior suspensions and then calculate the zeta potential for each at different pHs between 2 and 12 at 25 °C.

Both the surface area (m^2^g^− 1^) as well as the pore volume were measured using the Brunauer-Emmett-Teller (BET) and Barrett-Joyner-Halenda (BJH) techniques, respectively. The N_2_ isotherms generated by adsorption/desorption within the (Ag)Kao/ZnO-QDs nanocomposite under fixed sorption parameters at 77 K are acquired using a Micromeritics TriStar 3000 (Micromeritics Instrument Corporation, Norcross, GA, USA) with Quantachrome Touch Win Devices v1.11. Particles were heated to 200 °C for two hours before examination, and then outgassed to 10^− 3^ Torr using a Micromeritics FlowPrep™ station 060.

The ZnO and Ag_2_O nanostructures having optical properties (the band gap energy) in the produced (Ag)Kao/ZnO-QDs nanomaterial sample were examined using diffused reflectance spectra using UV-visible-NIR diffuse reflectance spectroscopy (DRS). This process occurred in the UV/VIS region among wavelengths 190 and 2500 nm with a resolution of 0.1 nm using the JASCO spectrometer (model V-570) [43].

### Photocatalytic degradation efficacy (Ag)Kao/ZnO-QDs photocatalyst utilizing IC and RhB dyes

During the summer months between 12:00 and 17:00 in Egypt (approx. latitude 31.4175° N, longitude 31.8144° E) when the temperature rose to about 30 °C under atmospheric pressure, the photocatalytic degradation effectiveness of the produced (Ag)Kao/ZnO-QDs nanocomposite was examined below solar irradiation to measure the dye removal development for Indigo Carmine (IC) and Rhodamine B (RhB) dyes in solution states. The sun’s intensity during the dye’s removal methods was measured using the Hl 97,500 Luxmeter which was determined to be 77 lx. Photocatalytic degradation tests were conducted using IC and RhB dyes in neutral, acidic and basal conditions with pH values of 7, 5, and 9, individually [44]. Furthermore, the dye elimination processes were actually carried out using various quantities of the doped silver through the Kao-Zn 15% nanostructure (1, 2, 3, 4 and 5% Ag) in order to ascertain the ideal ingredients of the (Ag)Kao/ZnO-QDs nanocomposite.

To determine RhB dye’s photocatalytic degradation capability at neutral solutions, this project firstly examined the influence of a constant initial dye concentration (10 mg/L) on varying quantities of photocatalyst ranging from 500 to 3000 mg/L. The effects of the 2500 mg/L optimal nanostructure quantity on various dyes ranging from 2 to 12 mg/L are additionally investigated. A 150 mL beaker was used in the pigment removing tests while 500–3000 mg/L of the photocatalyst were suspended in 100 mL of RhB pigment solution with a steady concentration of 10 mg/L. The suspensions were generated using various concentrations of photocatalyst then magnetically whiskered for half hour around 600 rpm within blackness in order to achieve the state of balance of dye adsorption/desorption. After that, the suspensions were left in sunlight about 150 min. Using the previously described methods for the IC dye in its neutral state and varying the initial dye concentration (30 mg/L) on varying amounts of nanocomposite (500–3000 mg/L). Secondly, by sustaining the optimum amount of nanostructure at 2000 mg/L over a variety of dye concentrations between 5 and 30 mg/L [45, 46].

The effect of the initial solution’s pH was investigated at acidic (pH = 5), neutral (pH = 7) and basic (pH = 9) statuses below the perfect circumstances for RhB dye (2500 mg/L of photocatalyst on 10 mg/L dye concentration) in order to achieve the most effective pH by employing sodium hydroxide (0.1 M) and hydrochloric acid (0.1 M). Next, by using the previously described methods under optimal IC dye circumstances (2000 mg/L photocatalyst on 30 mg/L dye concentration). On the other hand, so as to identify the most potent components of the (Ag)Kao/ZnO-QDs nanocomposite, the influence of different amounts of the doped silver through the Kao-Zn nanostructure were investigated against RhB and IC dyes under optimum conditions (1, 2, 3, 4 and 5% Ag) [47, 48].

In order to determine the effectiveness and extent of photodegradation process, tentative variations in dye concentrations were noted. Here, a 1.0 cm quartz cell and a UV-visible spectrophotometer (Jasco V-630, Japan) were used to quantify the absorption value. The λ_max_ values obtained from the absorption of IC and RhB dyes through UV-visible spectroscopy were 608 and 554 nm, individually. Following a certain amount of time (10 min), each 3 cm^3^ of dye dispersion was extracted and any hard nanocomposite were rapidly eliminated by centrifuging the model for ten minutes at 10,000 rpm. After that, each dye’s absorption rate had been evaluated and recorded.

The reduction of ^•^OH, e^−^, h^+^ and ^•^O_2_^−^ has been done to determine the mechanisms and types that are essential and efficient for the photocatalytic degradation process of IC and RhB dyes utilizing produced (Ag)Kao/ZnO-QDs nanocomposite exposed to sunlight. This occurred by adding 76.8 µL/L iso-propanol (^•^OH scavenger), 170 mg/L of silver nitrate (e^−^ scavenger), 166 mg/L potassium iodide (h^+^ scavenger) and 108 mg/L *P*-benzoquinone (^•^O_2_^−^ scavenger) individually to the reaction surroundings under the same previous optimal conditions for the IC and RhB dyes solution. Before being exposed to solar irradiation for additional an hour, each of the solutions that included the scavenger chemicals for each dye were placed in blackness in the same period which stirred by magnets that rotated about 600 rpm. Finally, for every previous experiment, 3 cm^3^ of each combination were set away at a specific time (10 min), quickly centrifuged for 10 min about 10,000 rpm to remove any rigid nanomaterial parts and each sample’s absorbance was measured with a UV-visible spectrophotometer [49, 50].

### Photodegradation findings analysis

UV spectrometers have been employed to obtain the absorbance worthies A_0_ and A_t_ for IC and RhB dyes at λ_max_ = 608, 554 nm, individually which corresponded to Beer-Lambert’s principle [43]. A_0_ and A_t_ have a frontal relationship with C_0_ and C_t_ for the original dye sample and the dye sample that has experienced photocatalytic degradation process after a certain duration of time (t), individually. As a result, it is significant that absorbance (A) may be converted to concentration (C) using a calibrated correlation for IC dye (R^2^ = 0.999), which is represented in Eq. (1) and Fig. S1.

1 $${\text{C}}\,=\,{\text{54}}.{\text{939 A }} - \,0.{\text{4111}}$$

It is also possible to demonstrate the calibrated connection for RhB dye (R^2^ = 0.999) using Eq. (2) and Fig. S2.

2 $${\text{C}}\,=\,{\text{4}}.{\text{9239 A}}\,+\,0.{\text{16}}0{\text{8}}$$

Equation (3) was employed to calculate the photocatalytic degradation rate (D%) using the data gathered for the previous dyes (under different dye concentrations, amount of Ag doped, pH and photocatalyst quantities):

3 $${\text{D }}\% {\text{ }}={\text{ }}\left( {{{\text{C}}_0}--{\text{ }}{{\text{C}}_{\text{t}}}} \right)/{{\text{C}}_0}{\text{x 1}}00$$

where C_0_ represents the initial concentration of each dye and C_t_ represents the final dye concentration following the dye removal process in the occurrence solar irradiation.

The elimination method of IC and RhB dyes by photocatalysis has been investigated using pseudo first-order kinetics. Equation (4) has been utilized to ascertain the photodegradation kinetics in order to establish the ideal photodegradation situations:

4 $${\text{ln }}\left( {{{\text{C}}_0}/{{\text{C}}_{\text{t}}}} \right)\,=\,{\text{kt}}$$

The slope in the linear area of the diagram of ln (C_0_/C_t_) versus (t) indicates the experimental variables used to determine the photodegradation rate constant (k, min^− 1^).

## Results and discussion

### Features of (Ag)Kao/ZnO-QDs

#### XRD microstructure and composition

**Fig. 1:**
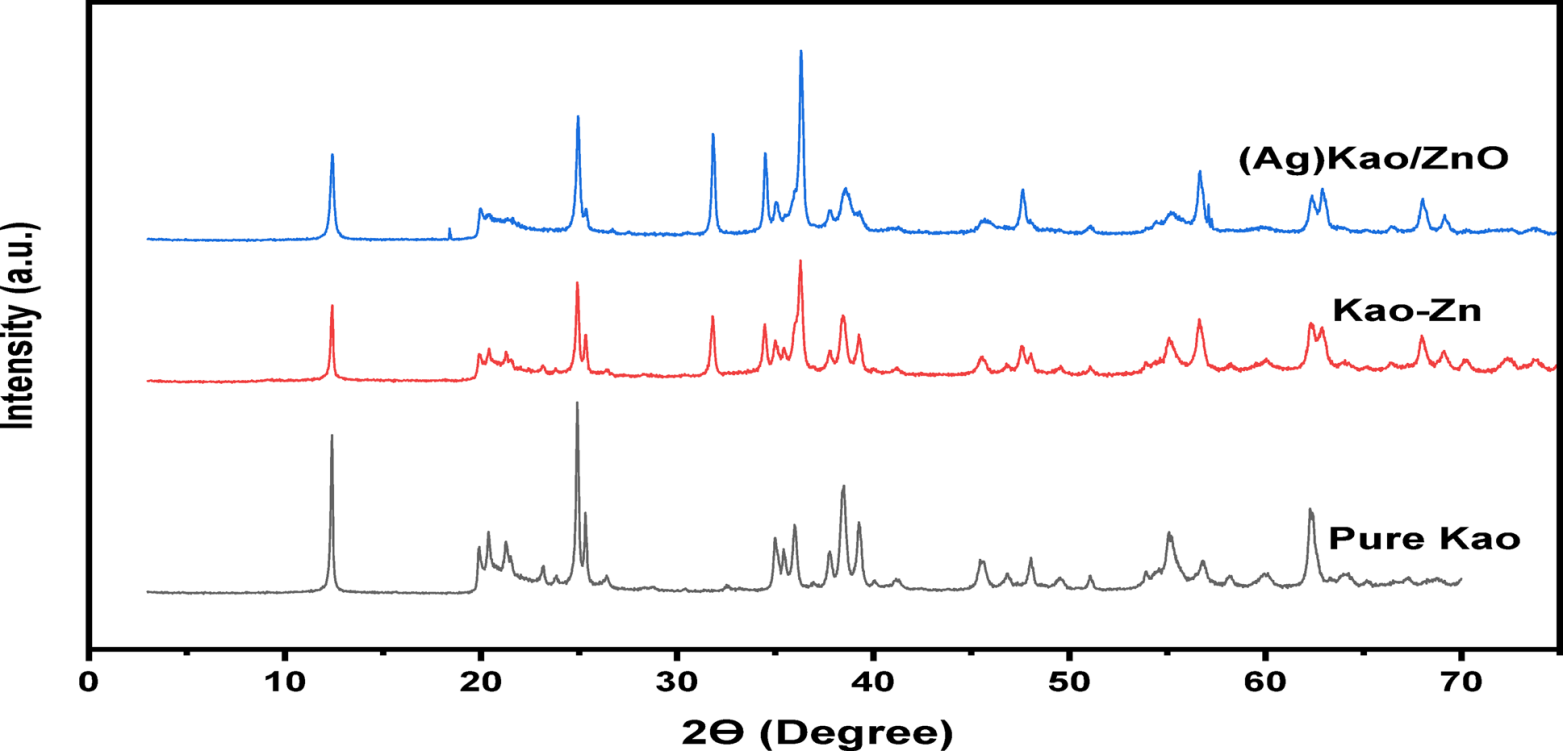
XRD scans of the models of pure kaolinite (Kao), Kao-Zn nanocomposite in addition (Ag)Kao/ZnO-QDs nanostructure

After mixing purified kaolinite with AgNO_3_ as well as Zn (NO_3_)_2_.6H_2_O to 400 °C over a speed of 10 min for each one ^o^C degree, all three X-ray diffractograms that compared with each other (Fig. 1) confirmed that the kaolinite was not suffering any form of transformation through the synthesized (Ag)Kao/ZnO-QDs nanostructure. XRD reflection bands between 10^o^ and 70^o^ 2θ showed that the phyllosilicate formula of kaolinite had been chemically stable. This happened because dehydroxylation limitation of kaolinite [Al_2_Si_2_O_5_(OH)_4_] had not been reached at the last heat of 400 °C within the preparation process however began at 450 °C. Comparing kaolinite with different clay minerals, it can be determined that it is the ideal clay used in this creation because of its essential heat constancy [51, 52] .

Analysis of the crystallite size D_001_ plus crystallinity index (HI) for clean kaolinite, Kao-Zn nanocomposite and the generated (Ag)Kao/ZnO-QDs nanostructure revealed that the latter two have specific microstructural modifications. According to Fig. 1, the Ag_2_O phases in the X-ray graph through the (Ag)Kao/ZnO-QDs nanostructure showed that this form of nanocrystallization took place at 2Ɵ equal 18^o^ (110) and 57^o^ (110).

#### X-ray photoelectron spectroscopy (XPS)

**Fig. 2:**
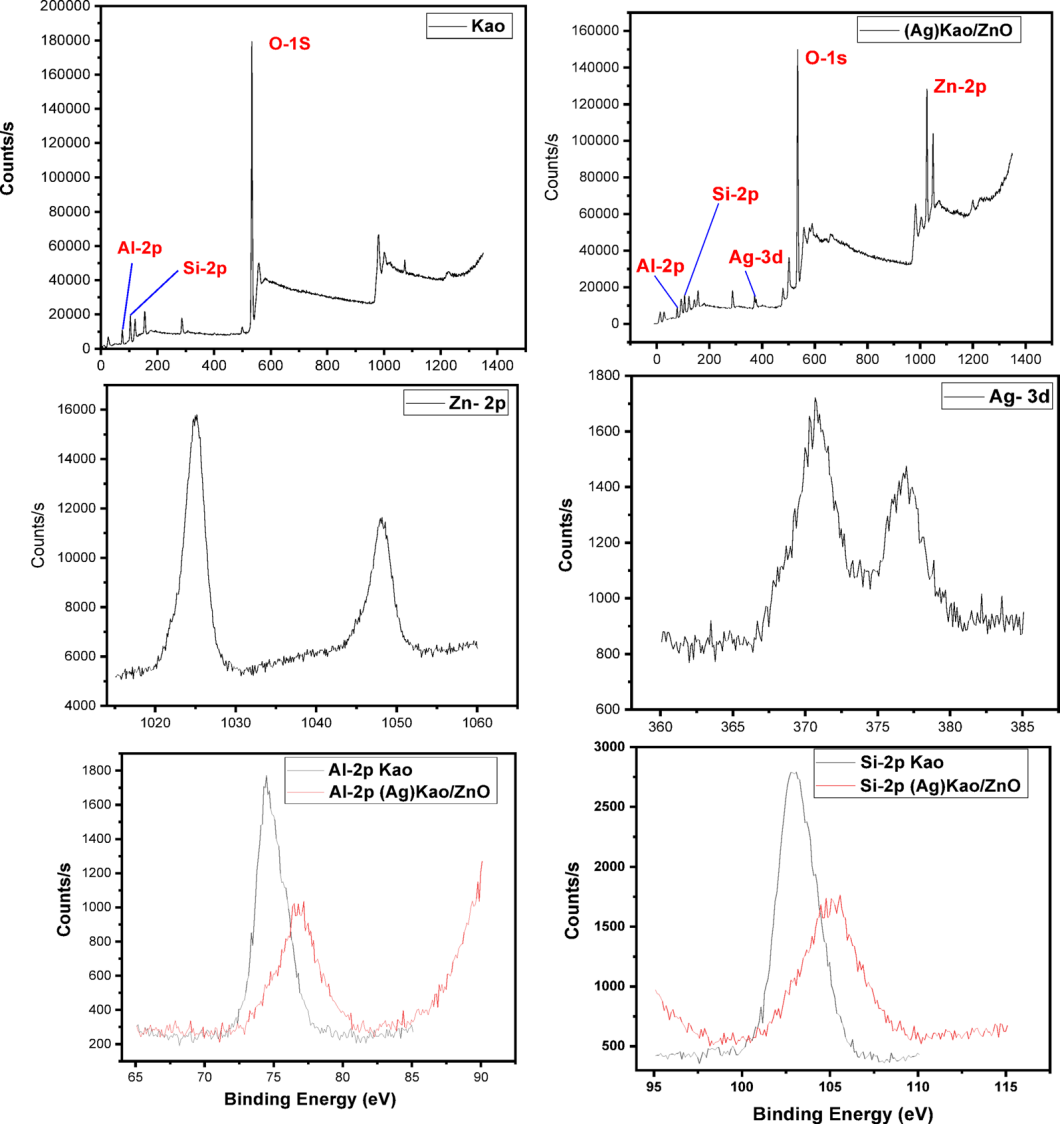
XPS scanning spectra of the pure kaolinite (Kao) as well as (Ag)Kao/ZnO-QDs nanostructure

The XPS analysis spectra for the clean kaolinite and (Ag)Kao/ZnO-QDs nanostructure in the 0–1200 eV range which contains the verified indicators of Zn, Ag, O, Al and Si was displayed in Fig. 2. From the data provided, 2 separated bands at 1025 and 1048 eV were visible in the higher resolution spectra of Zn electrons within the (Ag)Kao/ZnO-QDs nanocomposite. It was also observed two disconnected bands of Ag electrons at 371 and 377 through the (Ag)Kao/ZnO-QDs nanostructure. In addition, the (Ag)Kao/ZnO-QDs nanostructure exhibited lower signal strengths for Al, Si and O in the high-resolution spectra in contrast to the purified kaolinite particles. (Fig. 2). When compared to pure kaolinite peaks, the spectral patterns of these components revealed slightly different in peaks position throughout the (Ag)Kao/ZnO-QDs photocatalyst. The alterations in peaks sites and strengths demonstrate that ZnO plus Ag_2_O were doped across kaolinite sheets [53, 54].

#### Fourier transform infrared (FTIR) spectroscopy‏

**Fig. 3:**
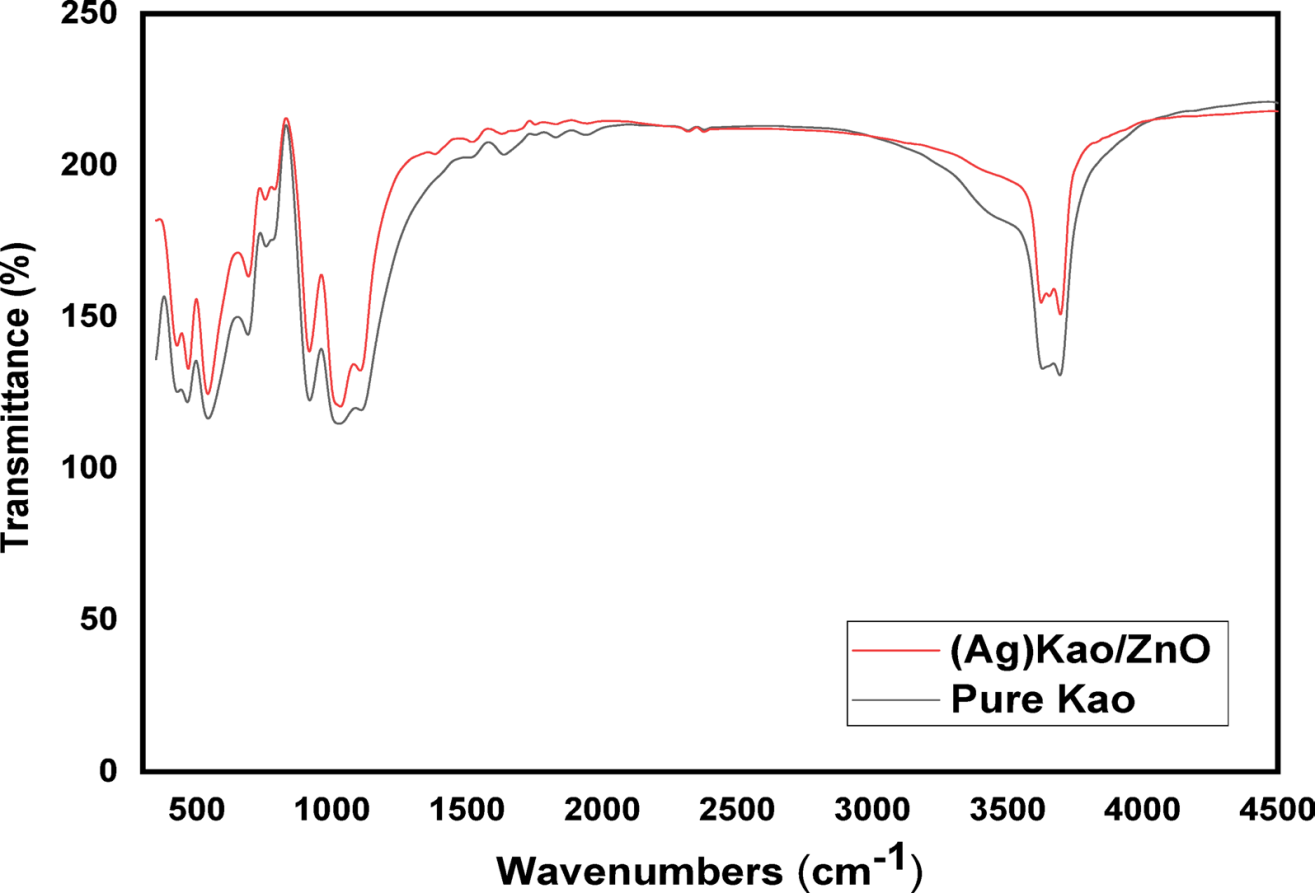
The FTIR investigations of the (Ag)Kao/ZnO nanostructure and pure kaolinite

Figure 3 displays the findings of a FTIR band investigation of pure kaolinite and the (Ag)Kao/ZnO-QDs nanostructure. Following silver and zinc are reacted across the superficial of kaolinite, FTIR analyses between 300 and 4500 cm^− 1^ regions can be used to detect changes in the OH stretching vibrations of the nanomaterial, particularly for peaks which appear in the 3500–3800 cm^− 1^ frequencies range. There were significant differences in the absorption amount of the OH-stretching vibrational bands between the pure kaolinite and (Ag)Kao/ZnO-QDs nanostructure FTIR spectra that emerged at 3620 and 3695 cm^− 1^. The peaks mentioned previously were linked to inner OH groups (Al-O-H) as well as edge-hanging OH chains (Al-O-H and Si-O-H). The dispersal of Ag and Zn atoms during the complete decomposition of AgNO_3_ and Zn (NO_3_)_2_.6H_2_O particles through kaolinite and their thermic expansion within the OH groups attached to the verge and inside of the molecule to create the (Ag)Kao/ZnO-QDs nanocomposite are the causes of these deviations [36, 55]. When contrasted with pure kaolinite, the (Ag)Kao/ZnO-QDs nanocomposite spectrum shows an amplification or new peak in the 430–470 cm⁻¹ range which confirming an existence of ZnO nanoparticles. Since they don’t create strong interactions with IR-active functional groups, silver (Ag⁰) nanoparticles usually don’t exhibit straight vibrational bands in FTIR. On the other hand, indirect proof of Ag inclusion can be determined by band shifts or variations in intensity in the OH area (3600–3700 cm⁻¹) and the Si–O region (1000–1100 cm⁻¹) may indicate interactions between Ag nanoparticles and the kaolinite structure or surface hydroxyls. Moreover, some interactions may be caused by Ag addition as indicated by small shift and enhanced intensity of the OH stretching zone.

#### Transmission electron microscope (TEM) and scanning electron microscope (SEM)

**Fig. 4:**
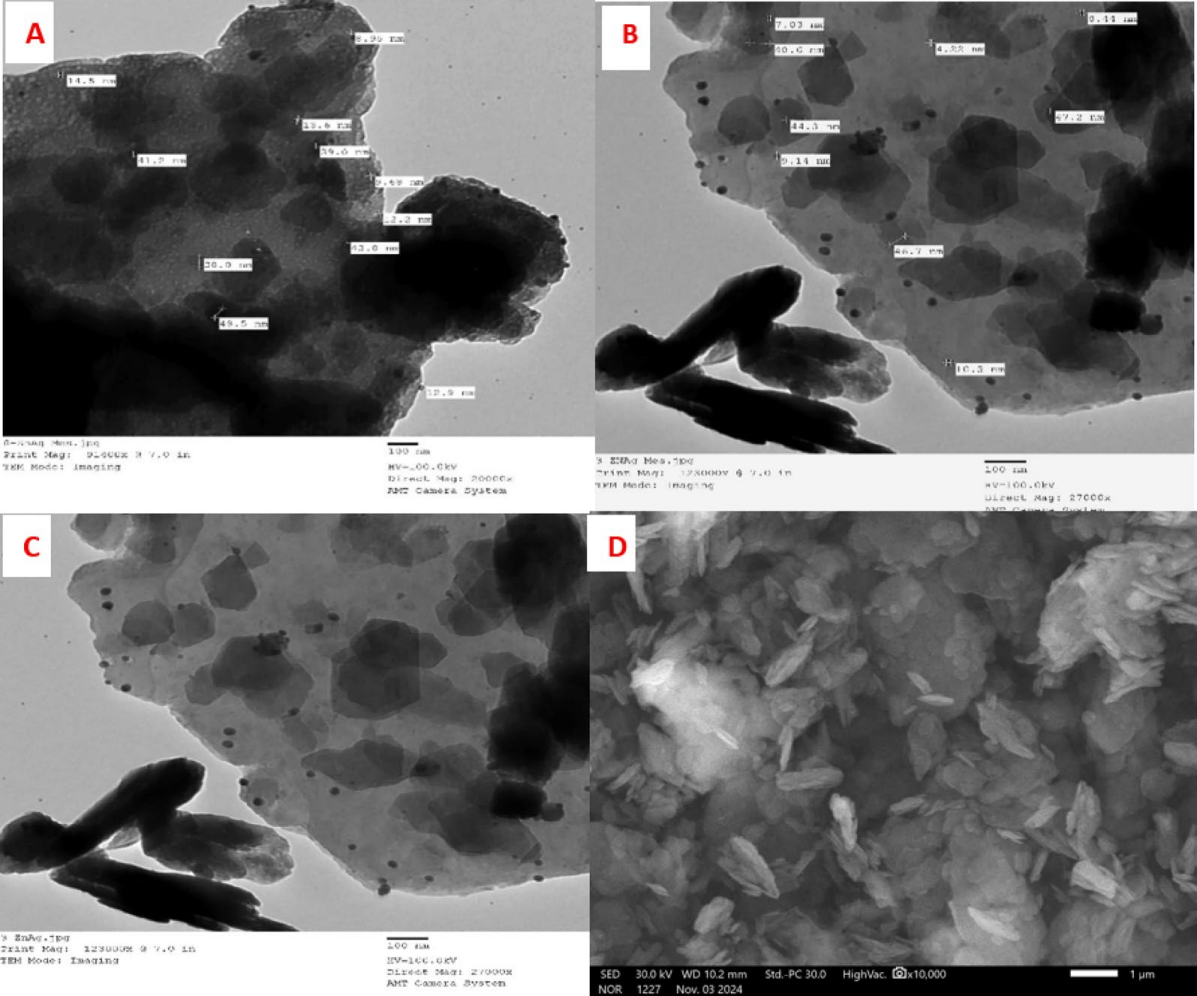
TEM and SEM of prepared (Ag)Kao/ZnO-QDs nanocomposite

**Fig. 5:**
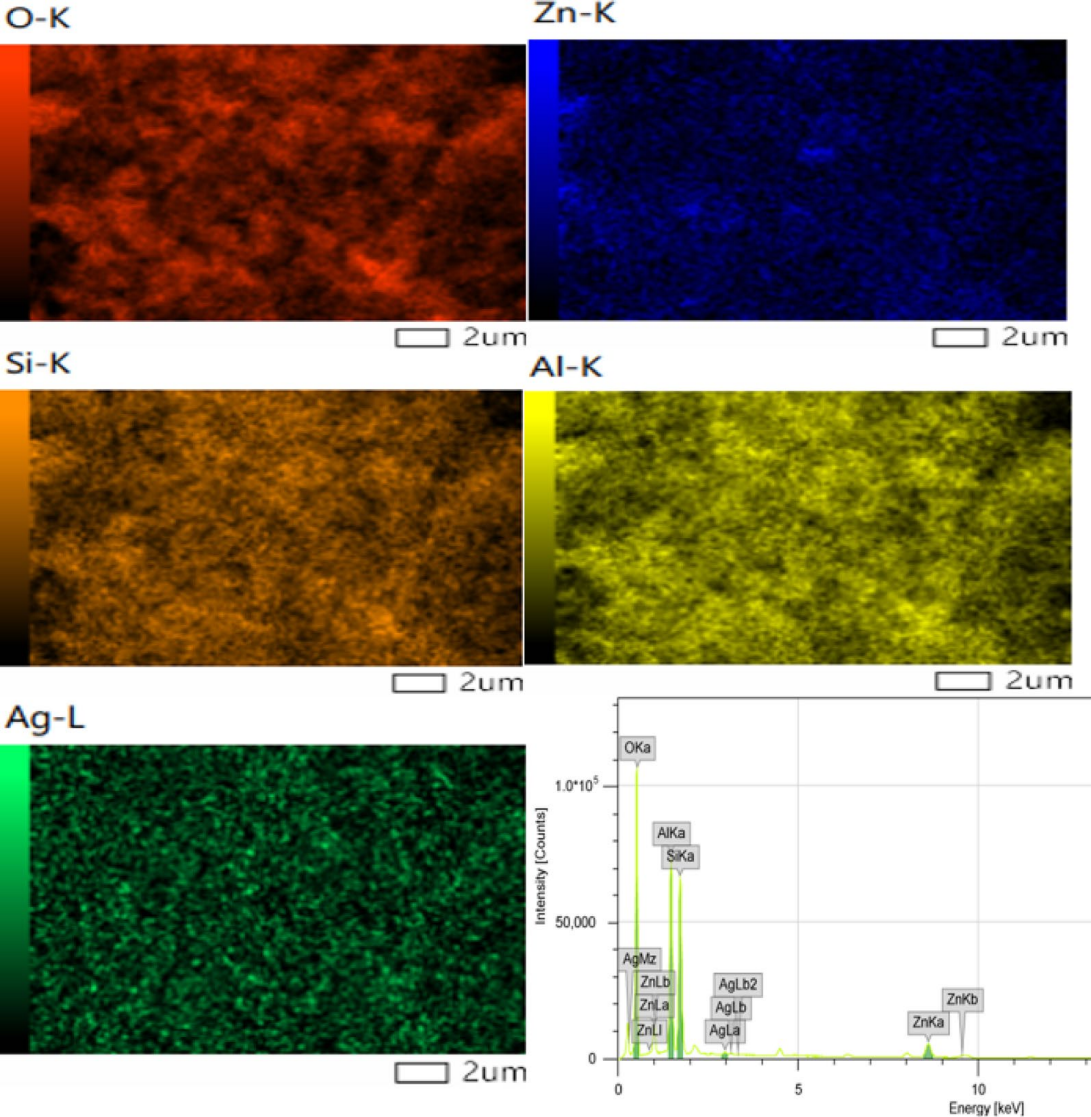
The EDX map and EDX components investigation of synthesized (Ag)Kao/ZnO-QDs nanoparticles.

Images from scanning and transmission electrons microscopy show that the finest crushed state of the (Ag)Kao/ZnO-QDs nanostructure units is composed of tiny combinations ranging from 500 nm to 1 μm of layered nanosheets ranging from 50 to 200 nm. Shown in Fig. 4, TEM investigations were used to identify the range of sizes of the zinc plus silver.

nanoparticles that were generated across (Ag)Kao/ZnO-QDs nanostructure. According to the TEM investigation, Ag and Zn atoms were effectively loaded onto the kaolinite interface to form (Ag)Kao/ZnO-QDs nanocomposite. In contrast, the (Ag)Kao/ZnO-QDs nanostructure’s EDX map which is displayed in Fig. 5 revealed that zinc in addition silver atoms was loaded as quantum dots which are smaller than 5 nm. These silver and zinc quantum dots were evenly spreaded on the border as well as basic surfaces within the kaolinite nanosheets. The produced (Ag)Kao/ZnO-QDs nanostructure’s EDX investigation revealed that the mass proportions of Si, Al, O, Zn, and Ag were 18.03, 16.93, 58.17, 5.99 and 0.88, respectively, as illustrated in Fig. 5 [56, 57].

#### Surface charge (zeta potential)

**Fig. 6:**
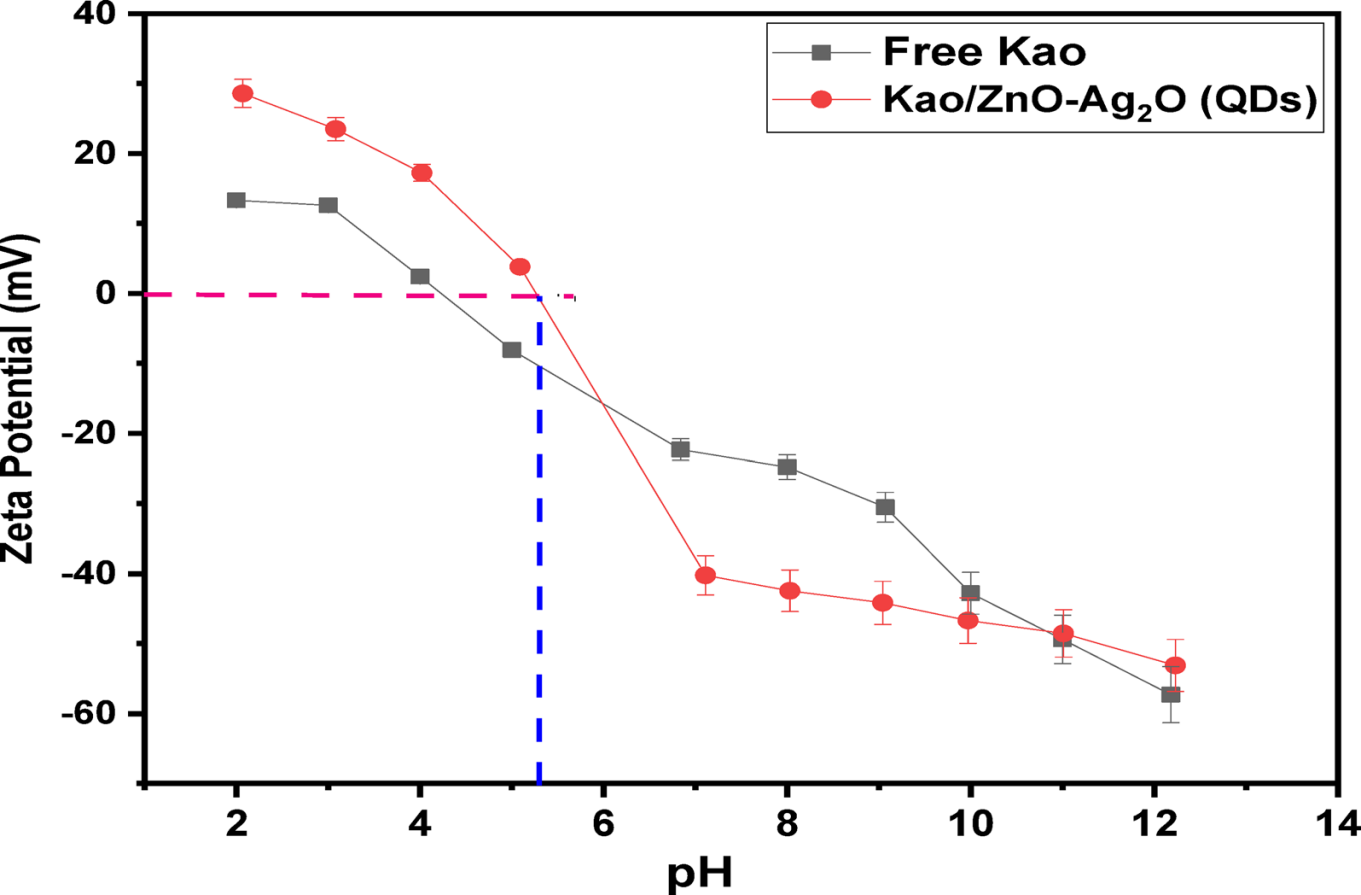
Zeta potential (ZP) outcomes of kaolinite free as well as the (Ag)Kao/ZnO-QDs nanostructure

Figure 6 displays the electrified charges on the outer layer of both free kaolinite and the (Ag)Kao/ZnO-QDs nanocomposite which depends on the analysis of the zeta potential using pH readings ranging from 2 to 12. At pH levels between 4.2 and 12.18, kaolinite commonly displays a permanent negative charge with the highest negative value of -57.3 mV at pH = 12. It changes to a positive state at pH values below 4.2. The point of zero charge (pzc) for purified kaolinite is found at pH = 4.2. Additionally, the generated (Ag)Kao/ZnO-QDs nanostructure’s pzc is identified at pH = 5.3 with an increase in the negatively charged out layer which approaches − 53.13 mV at pH = 12.23 when pH greater than 5.3. Otherwise in acidic conditions (pH < 5.3), the positive charge of the (Ag)Kao/ZnO-QDs nanostructure increases to + 28.6 mV at pH = 2.07 [58].

#### N_2_ adsorption/desorption studies

**Fig. 7:**
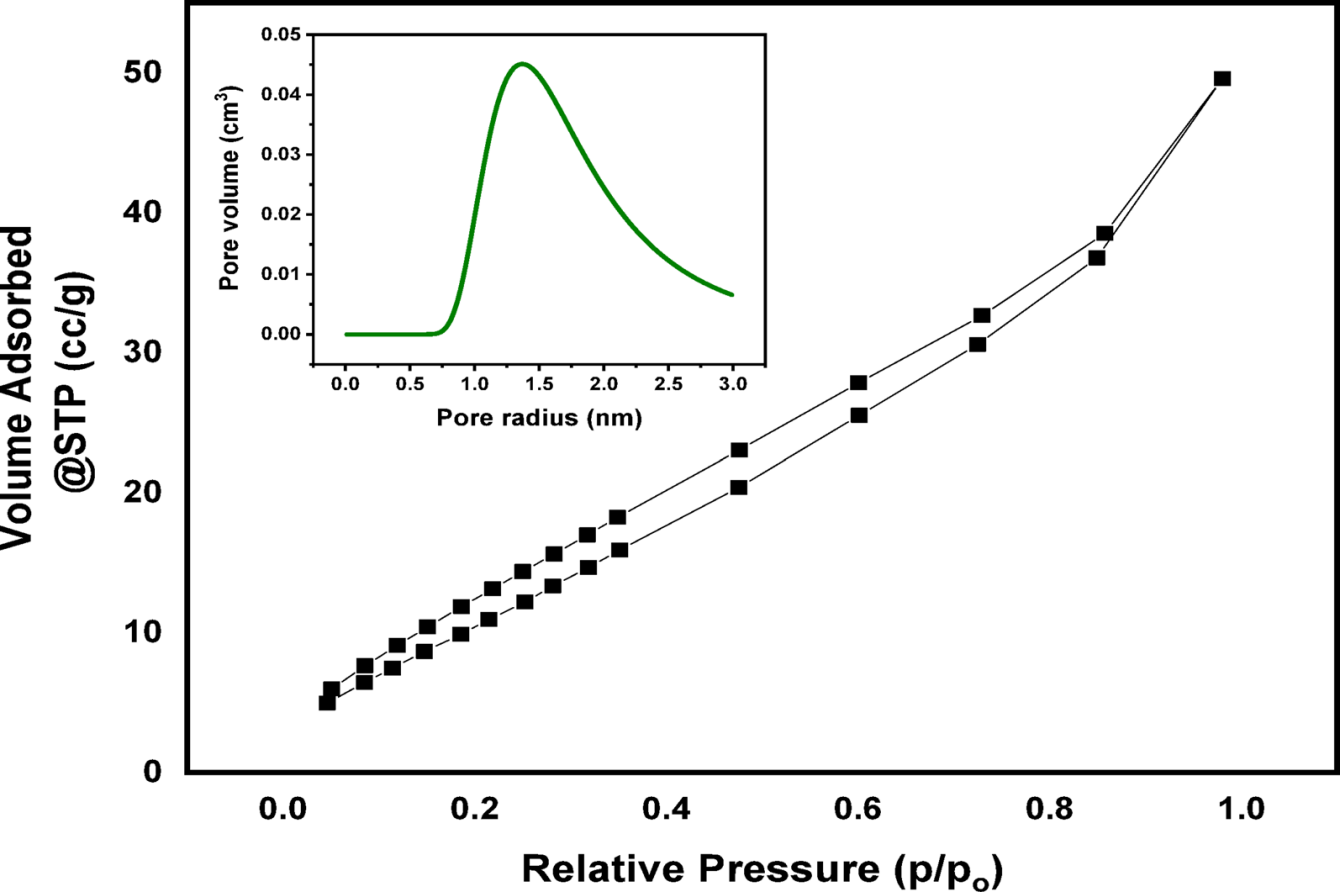
N_2_ adsorption/desorption isotherm for the (Ag)Kao/ZnO-QDs photocatalyst at 77 K and 400 °C

N_2_ adsorption/desorption measurements at 77 K were utilized to examine the Barrett-Joyner-Halenda (BJH) pore size and Brunauer-Emmett-Teller (BET) specified surface area of the (Ag)Kao/ZnO-QDs nanostructure. By using the BET formula in its typical range of uses, the value of 16.2 Å was selected for the N_2_ particle’s cross-sectional region shown Fig. 7. In comparison to the refined kaolinite model which has a surface area of 45.37 m^2^g^− 1^, the (Ag)Kao/ZnO-QDs nanocomposite has a slightly greater definite surface area of 50.3148 m^2^g^− 1^. Furthermore, as illustrated in Fig. 7, its BJH total pore volume reached 0.0769 cc/g while its BJH pore size under saturation pressure was 3.0565 nm [59]. Thus, the IUPAC classification describes the (Ag)Kao/ZnO-QDs nanostructure to be a mesoporous materials due to its pore size is greater than 2 nm. The modest hysteresis loop and significant rise at high relative pressures (P/P₀ >0.8) suggest a Type IV isotherm, which is typical of mesoporous materials (pore size between 2 and 50 nm).

#### Optical band gap energy (E_g_)

The nanostructure’s visual properties have been examined using UV-Vis diffuse reflectance spectroscopy (DRS). Using the acquired absorbance spectrum, the band gap energy (E_g_) of the (Ag)Kao/ZnO-QDs nanostructure was computed in accordance via Eq. (5):

5 $${{\text{E}}_{\text{g}}}={\text{ 124}}0/{{\text{l}}_{\text{g}}}$$

**Fig. 8:**
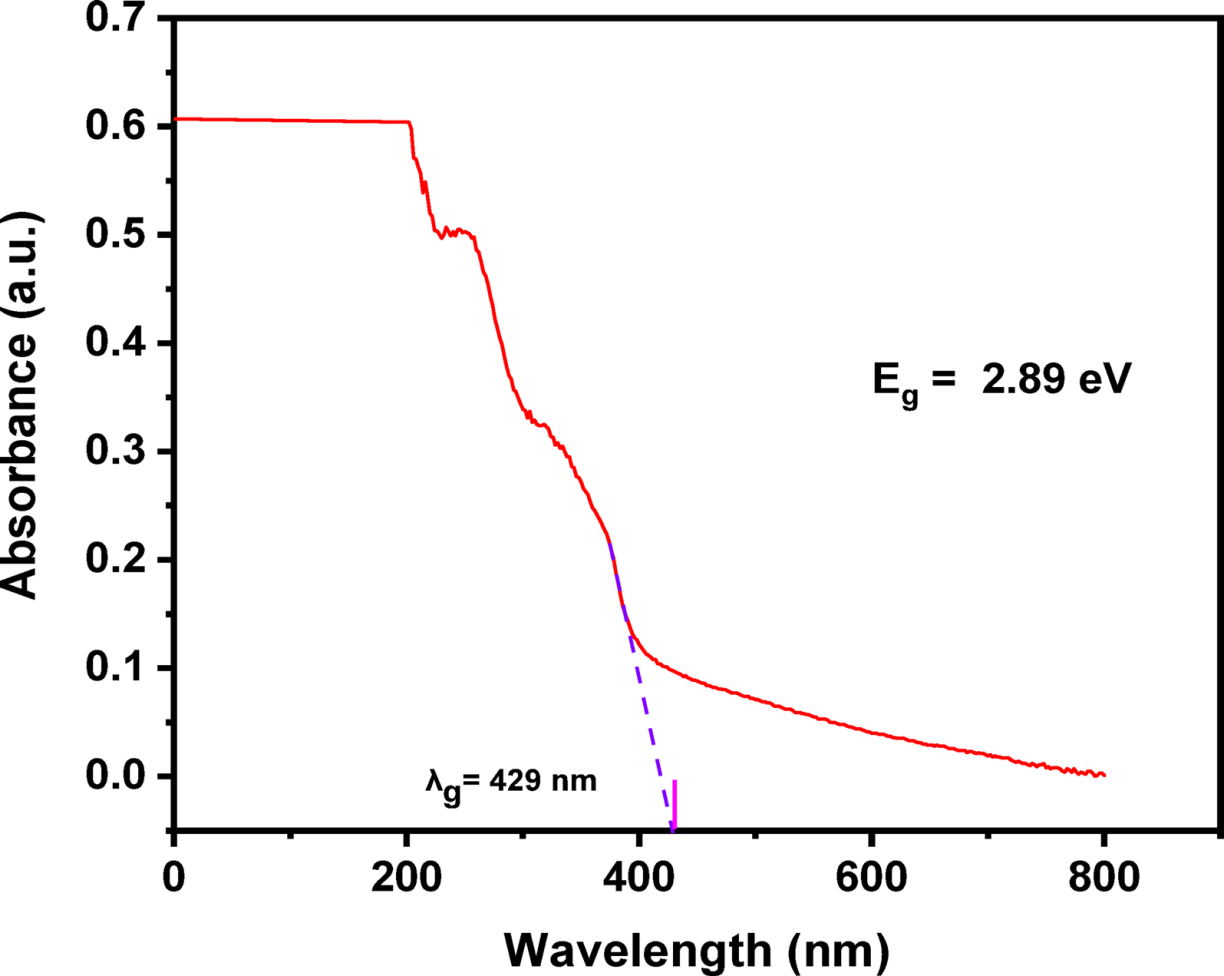
An investigation of the visual band gap energy of the (Ag)Kao/ZnO-QDs nanocomposite

In which λ_g_ referred to the absorbance wavelength and according to the diffuse reflected spectra in Fig. 8, λ_g_ was determined to be 429 nm. As a result, the estimated band gap energy of (Ag)Kao/ZnO-QDs nanostructure was 2.89 eV which appears to be significantly less than that of free kaolinite sample (6.2–8.2 eV) [60]. According to such decrease in band gap energy, doped Ag_2_O and ZnO over kaolinite surfaces improved electrons excitement from the least energy status (valence band) to the higher energy state (conducting band) which providing appropriate electrical conducting properties for the IC and RhB dye removal method (Fig. 8).

### The influence of operational factors on the IC and RhB dyes photocatalytic degradation methods

#### Effect of ag concentration

**Fig. 9:**
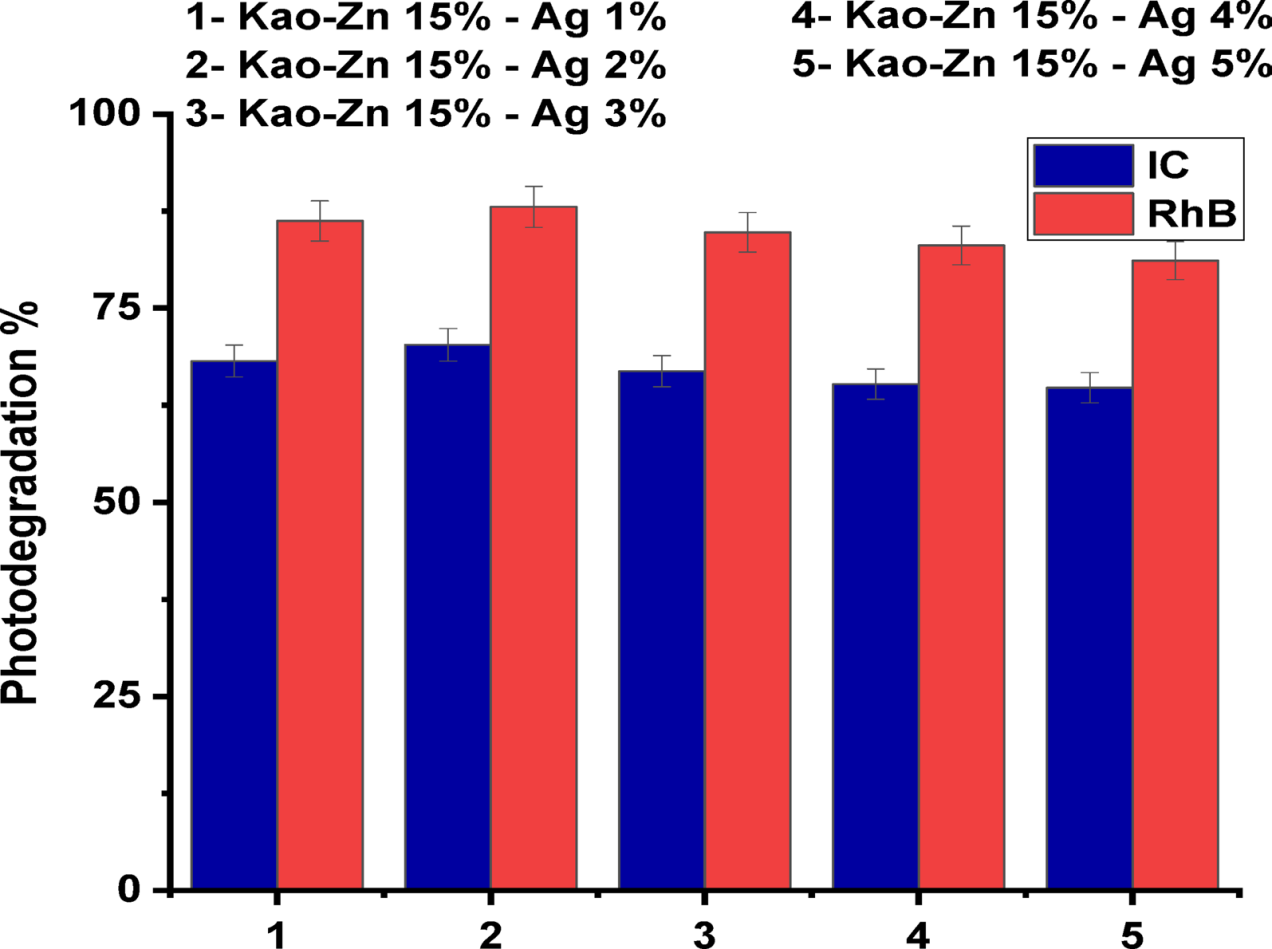
Effects of the quantities of Ag added at 400 °C to produce the (Ag)Kao/ZnO-QDs nanostructure on the photodegradation of IC (30 mg/L) as well as RhB (10 mg/L) dyes under neutral circumstances and direct sunlight

Figure 9 illustrates the effect of Ag percentage through the (Ag)Kao/ZnO-QDs nanostructure on the removal efficacy for IC and RhB dyes. The (Ag)Kao/ZnO-QDs nanocomposite was used to study the photocatalytic degradation procedure of IC and RhB dyes at varying Ag concentrations ranging from 1 to 5% Ag. The beginning concentrations of IC (30 mg/L) and RhB (10 mg/L) were used during these procedures about 90 min which was conducted for 30 min in darkness and 60 min below sunlight. Once the amount of Ag increases slightly in the (Ag)Kao/ZnO-QDs photocatalyst up to Ag 5%, the effectiveness of dye removal marginally growths till Ag 2% before it decreases. Therefore, (Ag)Kao/ZnO-QDs containing Ag 2% is the most efficient nanostructure for eliminating of IC and RhB dyes [61]. Generally speaking, higher Ag quantities can result lesser ^•^OH and ^•^O_2_^−^ in the IC colorant as well as lesser ^•^O_2_^−^ in the RhB colorant which reduce the removal performance. Nevertheless, when the amount of silver rose above 2%, bigger silver nanoparticles may additionally start to aggregate within the kaolinite’s surfaces. This may lead the silver particles to grow larger and reduce surface area which would decrease the rate of photodegradation procedure.

#### Influence of pH

**Fig. 10:**
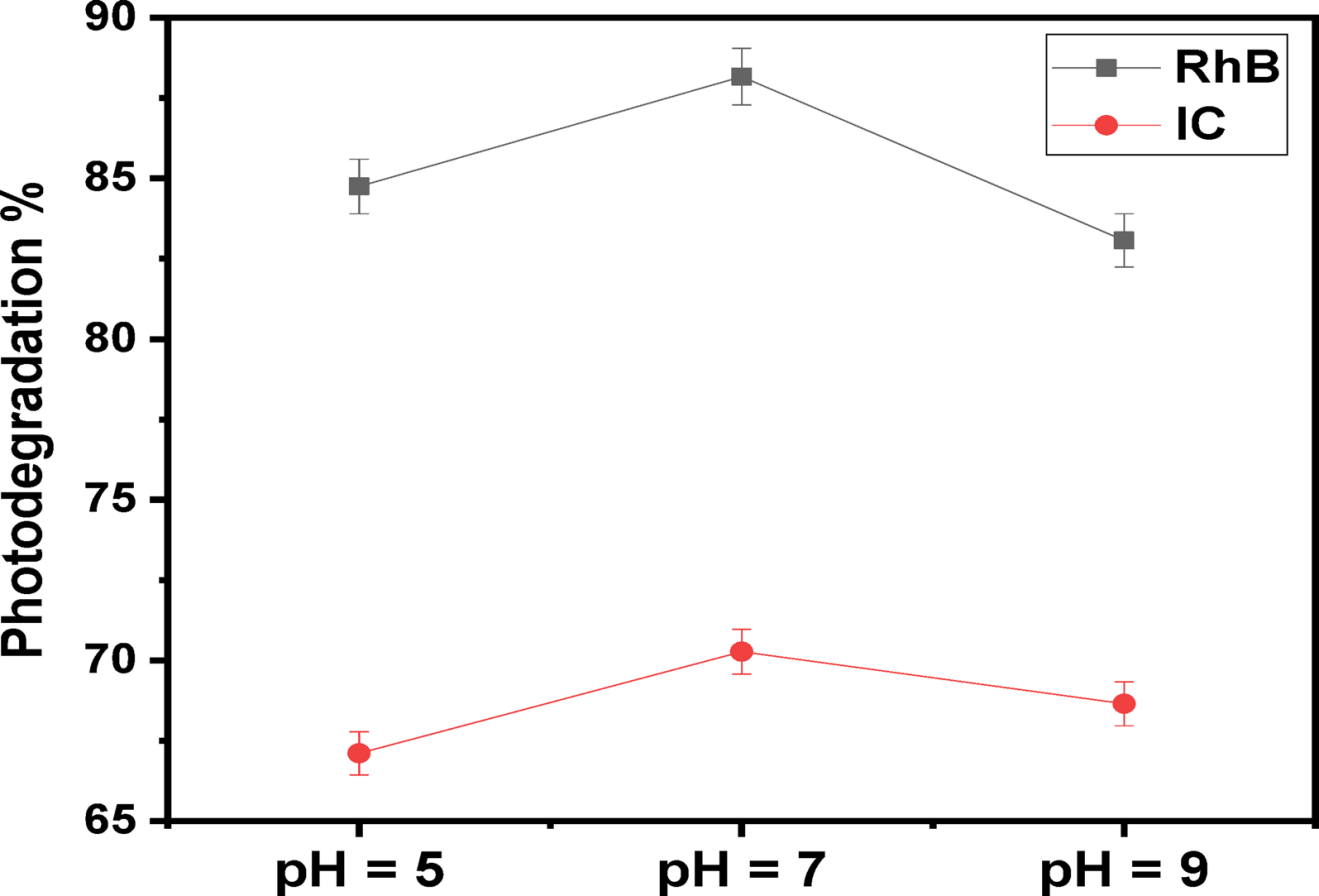
The influence of pH of IC (30 mg/L) as well as RhB (10 mg/L) dye solutions on the dye removal efficiency over (Ag)Kao/ZnO-QDs photocatalyst using direct sunlight

It is widely known in what way pH affects the ratio of the removal methods of various contaminated compounds. pH is a key operational factor that affects successfully on the photocatalytic degradation technique for removing a variety of contaminants from sewage. Figure 10 shows the performance in which the (Ag)Kao/ZnO-QDs nanostructure eliminates IC and RhB dyes at different pH levels in acidic (pH = 5), neutral (pH = 7) and basic (pH = 9) solutions which have been altered using sodium hydroxide and hydrochloric acid. The pH evaluation is conducted for the starting concentration of IC (30 mg/L) using (Ag)Kao/ZnO-QDs (2000 mg/L) plus RhB (10 mg/L) using (Ag)Kao/ZnO-QDs (2500 mg/L) following 30 min in the darkness and 60 min below solar energy. It was observed that the highest dye removal effectiveness for IC and RhB dyes was in neutral conditions (pH = 7) [62, 63].

#### Influence of photocatalyst quantity

**Fig. 11:**
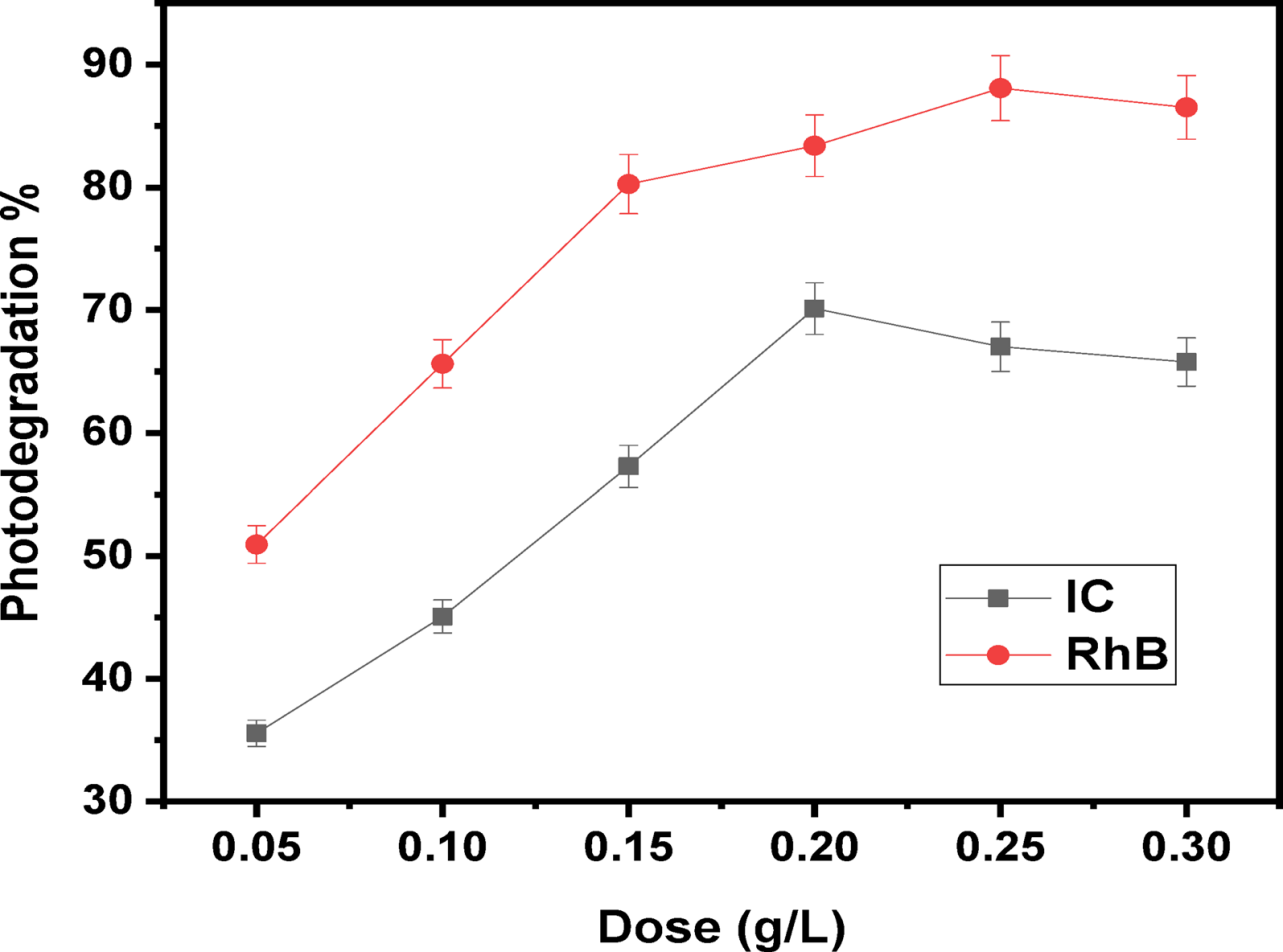
The contribution of (Ag)Kao/ZnO-QDs nanocomposite dosage on the removal rate of IC (30 mg/L) plus RhB (10 mg/L) at neutral solutions under solar irradiation

For excellent dye removing performance, it is evident that small amount of the nanocomposite increases the degree at which pairs of electrons and holes are produced which results in the generation of ^•^OH radicals [64]. Several quantities of (Ag)Kao/ZnO-QDs nanomaterial have been evaluated between 500 and 3000 mg/L range over fixed IC and RhB dye ratios of 30 and 10 mg/L, respectively with the aim to determine the optimal photocatalyst amount. The photocatalytic degradation measurements at neutral dye solution occurred for 30 min in darkness following an hour of exposure to sunlight are displayed in Fig. 11. As shown in Fig. 11, it became apparent that raising the nanostructure dose to 2000 and 2500 mg/L for IC and RhB dyes, separately progressively increased the dye removal rate prior it decreased. This happened because there were active sites on the (Ag)Kao/ZnO-QDs nanostructure surface. The rise in catalytic particles encouraged the creation of pairs of electrons-holes and generated a significant quantity of hydroxyl radicals. The efficacy in removing dyes became thereafter significantly reduced whenever the amount of (Ag)Kao/ZnO-QDs nanocomposite rose above the previous values [65, 66]. This could’ve happened because the nanostructure’s molecules gathered with each other that prevented photons from getting to the interior surface. Additionally, the high concentration of photocatalyst nanoparticles may be contributing in poor lighting transference as well as dispersed light in the supplied dye.

#### Influence of dye concentration

**Fig. 12:**
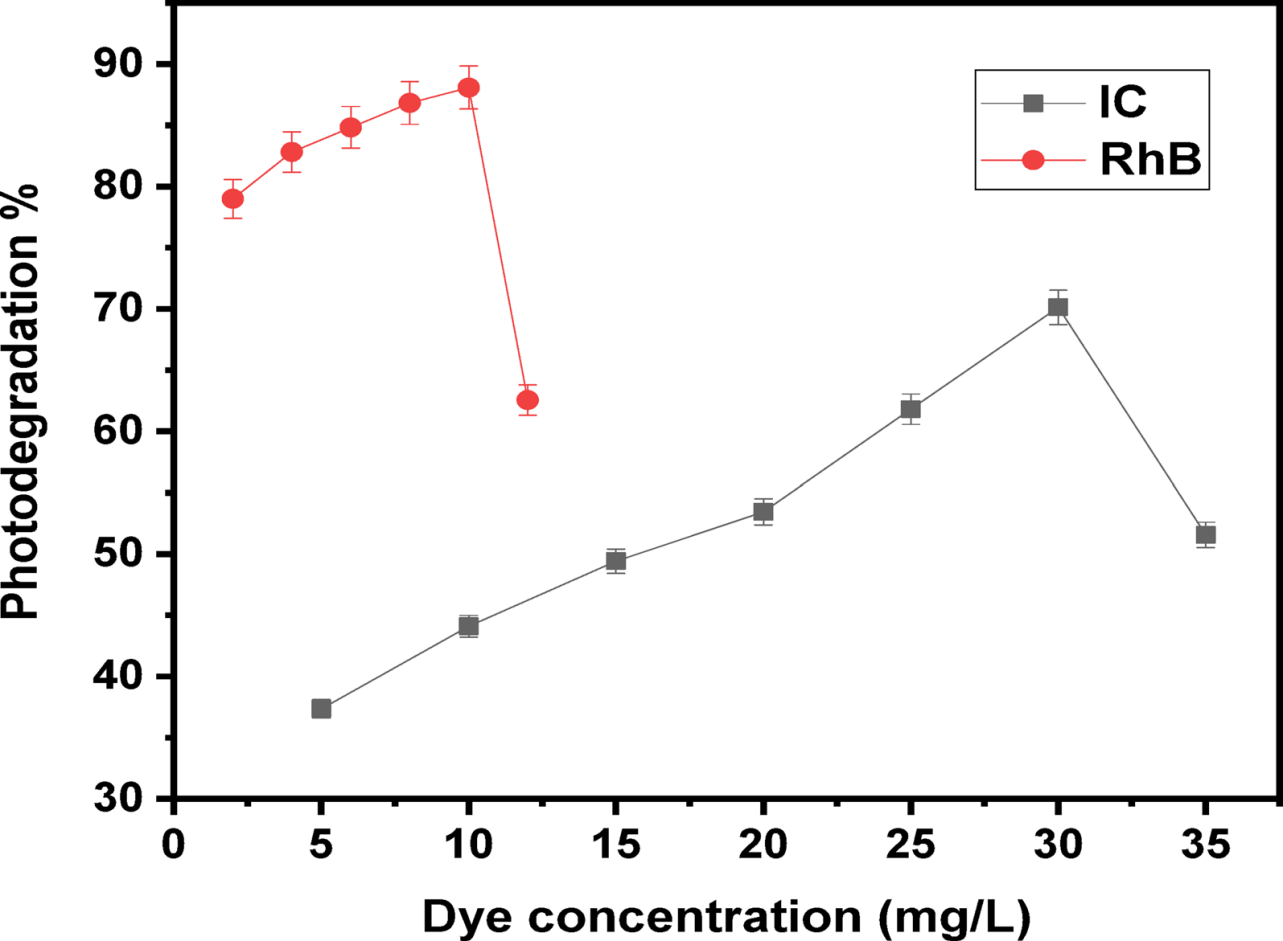
The contribution of IC and RhB dye ratios on the photocatalytic degradation efficacy of (Ag)Kao/ZnO-QDs nanostructure at neutral solutions with constant doses of 2000 and 2500 mg/L, individually under solar irradiation

The impacts of initial IC and RhB dye ratios on photodegradation process were examined using various dye ratios experiments. Throughout exposition to solar light, ratios of IC and RhB dyes from 5 till 35 mg/L and 2 till 12 mg/L have been studied using steady amounts of (Ag)Kao/ZnO-QDs nanocomposite 2000 and 2500 mg/L, individually. The optimal dye concentration for every dye was obtained using that approach in neutral conditions for an hour, as shown in Fig. 12. It was found that the dye removing ratios using (Ag)Kao/ZnO-QDs photocatalyst improved primarily for dye concentrations till 30 mg/L for IC and 10 mg/L for RhB before it declined. When the initial dye concentrations increased over the optimal previous values, the amount of ^•^OH radicals produced upon the photocatalyst interface dropped. This happened as a result of more dye molecules were absorbed onto the outer layer of the (Ag)Kao/ZnO-QDs nanostructure resulting in the decrease of photons absorbance. Moreover, the nanostructure reduced the number of photons were absorbed since the photons were stopped before they reached its surface [67, 68].

### Kinetics of photodegradation process

**Fig. 13:**
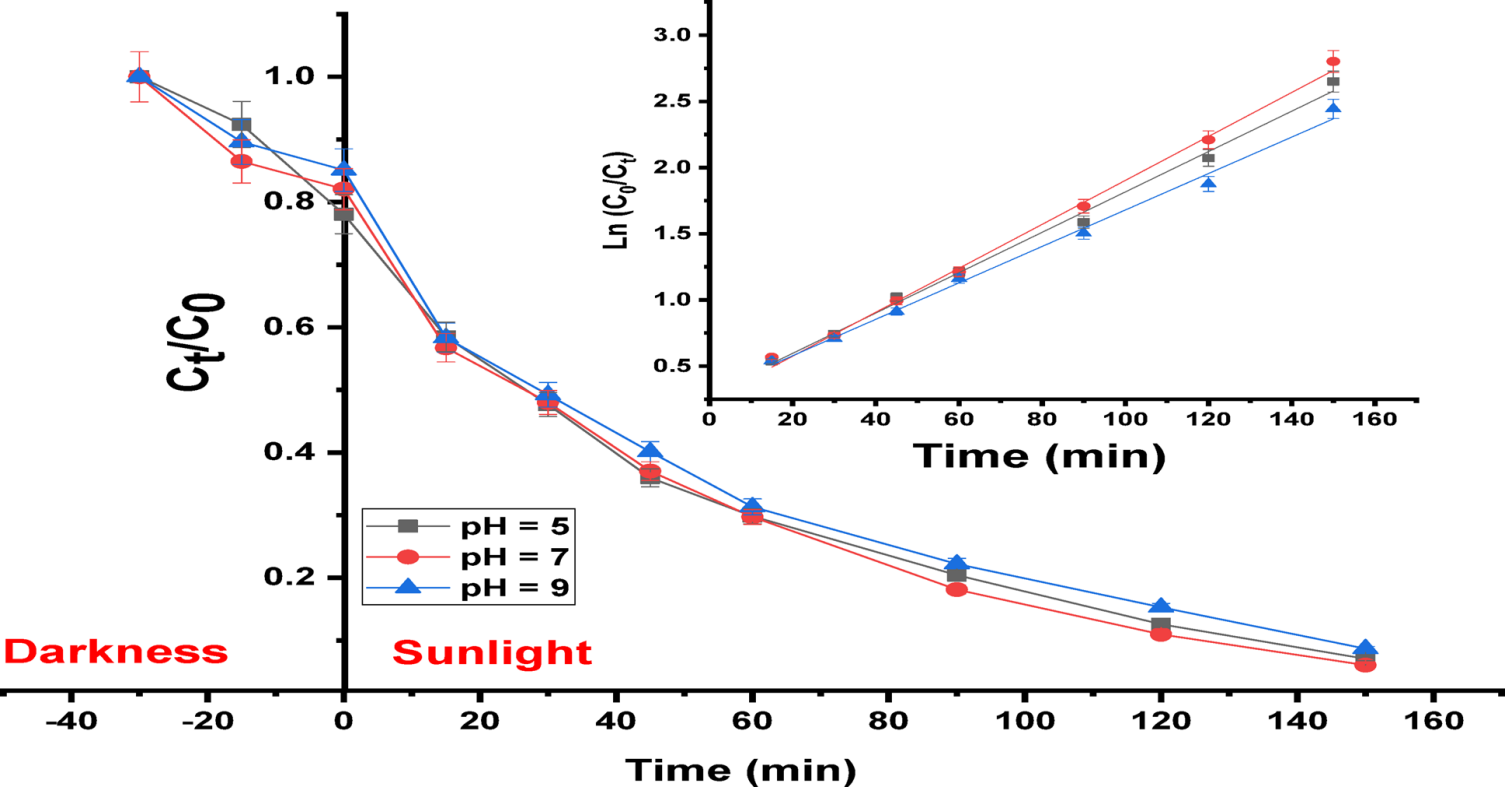
Kinetics of IC dye Photodegradation utilizing (Ag)Kao/ZnO-QDs nanostructure (2000 mg/L amount of photocatalyst with 30 mg/L dye ratio through pHs equal 5, 7, and 9) bared to sunlight

The dye removal efficacy of the prepared (Ag)Kao/ZnO-QDs photocatalyst below solar irradiation was evaluated utilizing IC and RhB dyes as simulation dyes. The kinetics of IC dye decomposition utilizing a (Ag)Kao/ZnO-QDs nanocomposite under different pH levels are displayed in Fig. 13. Once the relationship among ln (C_0_ / C_t_) vs. time was established, it proved that the IC dye degradation procedure matches precisely with pseudo-first-order kinetics. So, it is possible to determine the kinetic rate constant (k). It was discovered that the rate constants (k) readings for the various IC dye solutions with pHs equivalent 5, 7 and 9 were 0.0152, 0.0166 and 0.0138 min^− 1^ through correlation coefficients (R^2^) of 0.995, 0.997 and 0.995, respectively [69].

**Fig. 14:**
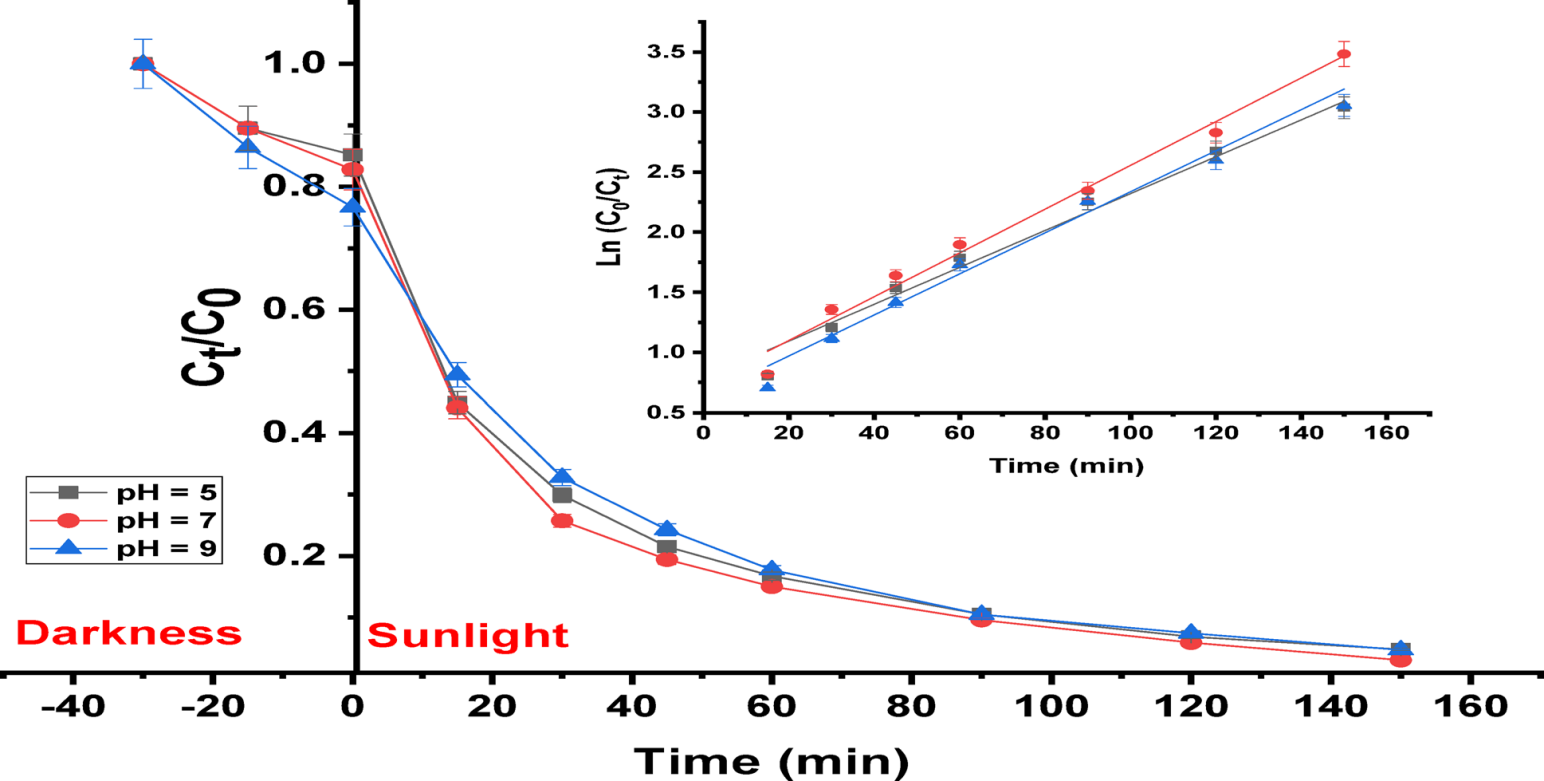
Kinetics of RhB dye Photodegradation utilizing (Ag)Kao/ZnO-QDs nanostructure (2500 mg/L amount of photocatalyst with 10 mg/L dye ratio through pHs equal 5, 7, and 9) bared to sunlight

On the other hand, the kinetics of RhB dye decomposition utilizing a (Ag)Kao/ZnO-QDs nanocomposite under changed pH levels are displayed in Fig. 14. After the relationship among ln (C_0_ / C_t_) vs. time was established, it proved that the RhB dye degradation procedure matches precisely with pseudo-first-order kinetics. So, it is possible to determine the kinetic rate constant (k). It was discovered that the rate constants (k) readings for the various RhB dye solutions with pHs equivalent 5, 7 and 9 were 0.0161, 0.018 and 0.0169 min^− 1^ through correlation coefficients (R^2^) of 0.984, 0.987 and 0.985, respectively [70].

### Mechanism of photodegradation process

**Fig. 15:**
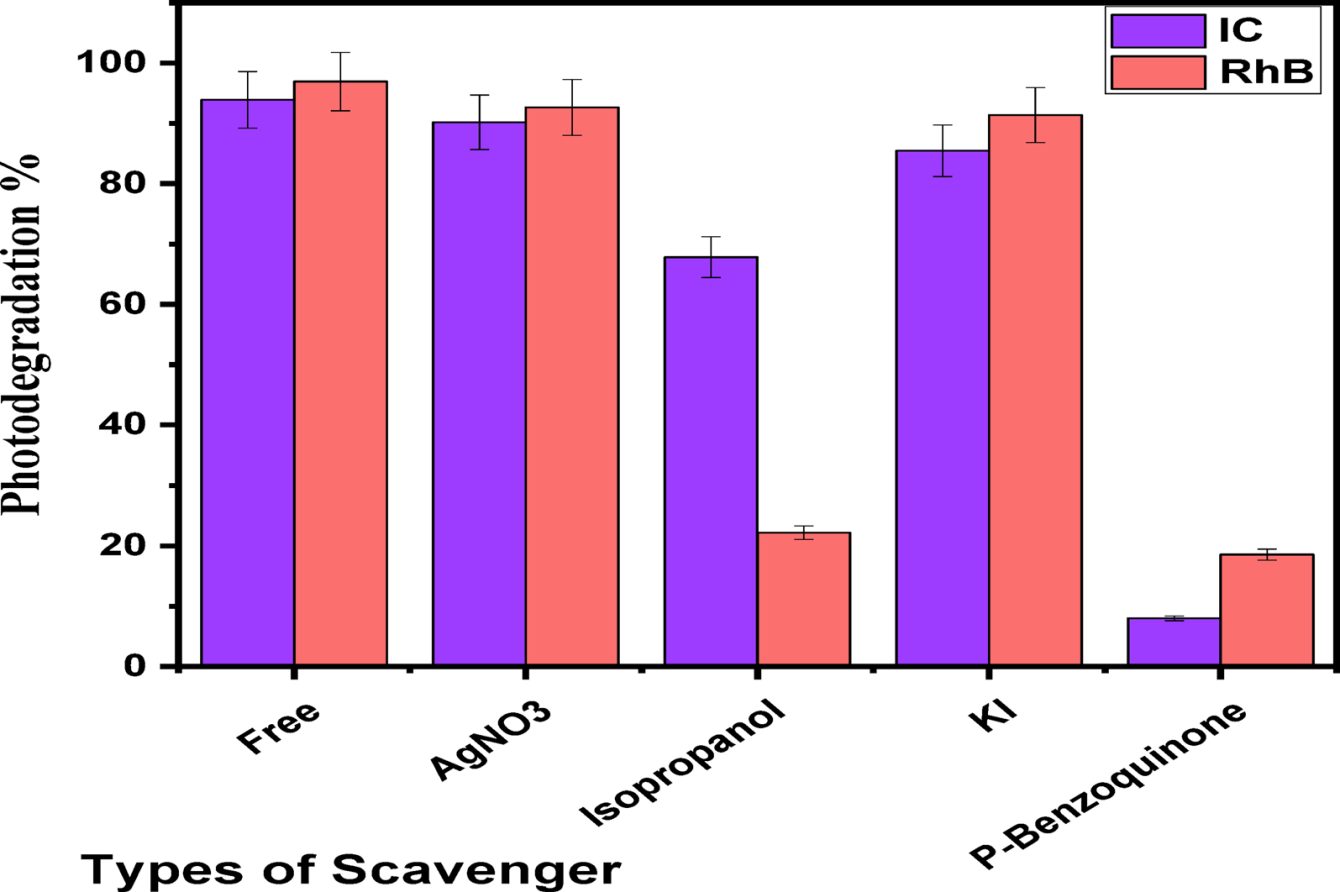
Ratios of IC and RhB dye photocatalytic degradation utilizing (Ag)Kao/ZnO-QDs nanomaterial prepared at 400 °C below direct sunlight in optimal circumstances without and with a variety of scavengers (silver nitrate, iso-propanol, potassium iodide and *p*-benzoquinone)

The photocatalytic degradation method consists of 2 primary levels: dye adsorption across the nanostructure interface and dye declination. Therefore, increasing the band gap energy, altering the crystalline construction and increasing the surface area may improve the (Ag)Kao/ZnO-QDs nanostructure’s dye removal efficiency [71]. Once nanomaterial exposed to solar energy, it often produces the reacting species e^–^, ^•^OH, h^+^ and ^•^O_2_^−^. To understand how the (Ag)Kao/ZnO-QDs nanostructure contributes to dyes decomposition, it is vital to identify the reacting species that are largely in control of the dye removal process by radical trapped (scavenger) investigations. Therefore, the dye removal process of IC and RhB dyes utilizing (Ag)Kao/ZnO-QDs was examined by adding silver nitrate (e^–^ scavenger), iso-propanol (^•^OH scavenger), potassium iodide (h^+^ scavenger) and *P*-benzoquinone (^•^O_2_^−^ scavenger) to the dye solutions as shown in Fig. 15 [72].

It was demonstrated that silver nitrate, potassium iodide and isopropanol had minimal effects on the photodegradation efficiency of IC dye after it had been broken down across (Ag)Kao/ZnO-QDs nanostructure below sunlight as shown in Fig. 15. The addition of *p*-benzoquinone (^•^O_2_^−^ scavenger) to IC dye solution was occurred greatly decrease in dye removal efficacy up to 7.98%. Consequently, superoxide radicals (^•^O_2_^−^) were the most reacting species during the decomposition process of IC dye utilizing the (Ag)Kao/ZnO-QDs nanoparticle under investigation. Thus, excited electrons (e^–^) from the valence band (VB) migrated to the conductive band (CB) leaving comparable holes (h^+^) in the nanostructure’s VB.

6 $$ \begin{gathered} \left( {{\text{Ag}}} \right){\text{Kao}}/{\text{ZnO}} - {\text{QDs}} + ~{\text{h}}\upsilon {\text{ }} \to {\text{ }}\left( {{\text{h}}^{{\text{ + }}} _{{\left( {{\text{VB}}} \right)}} } \right) + \left( {{\text{e}}^{{\text{ - }}} _{{\left( {{\text{CB}}} \right)}} } \right) \hfill \\ \hfill \\ \hfill \\ \end{gathered} $$

where the hʋ represents the power required to move just one electron from the VB to the CB.

7 $$ {\text{h}}^{{\text{ + }}} + {\text{ OH}}^{{\text{ - }}} {\text{or}}~^{~} ~{\text{H}}_{{\text{2}}} {\text{O}} \to ^{ \cdot } {\text{OH}} $$

8 $${{\text{O}}_{\text{2}}}\,+\,{{\text{e}}^ - }{ \to ^ \cdot }{{\text{O}}_{\text{2}}}^{ - }$$

The produced power is greater than the (Ag)Kao/ZnO-QDs nanoparticle’s band gap worth that is 2.89 eV. This facilitates the production of conducting band electrons (e^−^) and valance band holes (h^+^). The holes produced by the photocatalytic degradation process can not only instantly oxidize adsorbed IC dye but can additionally combine with hydroxyl groups (OH^−^) or H_2_O to generate hydroxyl radicals (^•^OH). When oxygen (O_2_) molecules are produced within outermost layer of the (Ag)Kao/ZnO-QDs nanocomposite, the electrons converted it to superoxide radicals (^•^O_2_^−^) [73, 74]. Ultimately, the resulting ^•^O_2_^−^ and ^•^OH largely decomposed the IC dye units generating CO_2_ besides H_2_O in the manner described below:

9 $${\text{IC }}{+^ \cdot }{{\text{O}}_{\text{2}}}^{ - }{/^ \cdot }{\text{OH}} \to {{\text{H}}_{\text{2}}}{\text{O}}\,+\,{\text{C}}{{\text{O}}_{\text{2}}}$$

On the other hand, it was demonstrated that silver nitrate besides potassium iodide had a small impact on the removal efficiency of RhB dye when it was photodegraded utilizing a (Ag)Kao/ZnO-QDs nanoparticle as shown in Fig. 15. Additionally, the dye removal rate was considerably decreased to 18.56% and 22.18% by adding *p*-benzoquinone (^•^O_2_^−^ scavenger) as well as iso-propanol (^•^OH scavenger), respectively. Based on this, hydroxyl radicals (^•^OH) besides superoxide radicals (^•^O_2_^−^) were the most reacting species throughout the RhB dye decomposition process that was being studied. Consequently, the valence band (VB) electrons (e^–^) of the (Ag)Kao/ZnO-QDs nanostructure got reactive and jumped to the conductive band (CB) leaving the VB containing equivalent holes (h^+^).

10 $$ \begin{gathered} \left( {{\text{Ag}}} \right){\text{Kao}}/{\text{ZnO}} - {\text{QDs}} + ~{\text{h}}\upsilon {\text{ }} \to {\text{ }}\left( {{\text{h}}^{{\text{ + }}} _{{\left( {{\text{VB}}} \right)}} } \right) + \left( {{\text{e}}^{{\text{ - }}} _{{\left( {{\text{CB}}} \right)}} } \right) \hfill \\ \hfill \\ \hfill \\ \end{gathered} $$

11 $$ {\text{H}}^{{\text{ + }}} ~ + {\text{e}}^{{\text{ - }}} + {\text{O}}_{{\text{2}}} ~ \to {\text{H}}_{{\text{2}}} {\text{O}}_{{\text{2}}} $$

12 $${{\text{H}}_{\text{2}}}{{\text{O}}_{\text{2}}}\,+\,{{\text{e}}^ - }{ \to ^ \cdot }{{\text{O}}_{\text{2}}}^{ - }+{\text{ O}}{{\text{H}}^ - }$$

13 $${{\text{H}}_{\text{2}}}{\text{O }}{+^ \cdot }{{\text{O}}_{\text{2}}}^{ - }{ \to ^ \cdot }{\text{OH}}\,+\,{\text{O}}{{\text{H}}^ - }$$

14 $$ {\text{OH}}^{ - } + ~{\text{h}}^{ + } \to ~^{ \cdot } {\text{OH}} $$

Later, the conductive band electrons interact with O_2_ to form ^•^OH and ^•^O_2_^−^ radicals accordingly. In addition, the h^+^ may produce additional ^•^OH which assisted in the RhB dye removal process. Because it has a minimal influence on the creation of ^•^OH and does not represent the main mechanism of RhB decomposition method, we cannot dismiss the indirect influence of ^•^O_2_^−^ on the photodegradation procedure of RhB dye based on the outcomes of the scavenger experiments. Perhaps the ^•^O_2_^−^ is just participating in a sideways reaction that has the potential to produce further ^•^OH for the RhB dye photodegradation procedure. The ^•^OH can also be produced by oxidizing h^+^ in VB the OH^−^ produced from water ionization that represents the main efficient species for RhB dye removal [75, 76]. Ultimately, ^•^O_2_^−^ and ^•^OH decomposed RhB dye which demonstrate that ^•^OH is an essential step in the dye removal mechanism. Following some time, such powerful radicals can decompose the RhB dye units releasing H_2_O and CO_2_:

15 $$ ^{ \cdot } {\text{O}}_{{\text{2}}} ^{ - } + ^{ \cdot } {\text{OH}} + {\text{ h}}^{ + } + {\text{RhB}} \to {\text{ CO}}_{{\text{2}}} + {\text{ H}}_{{\text{2}}} {\text{O}} $$

### Reusability and constancy

**Fig. 16:**
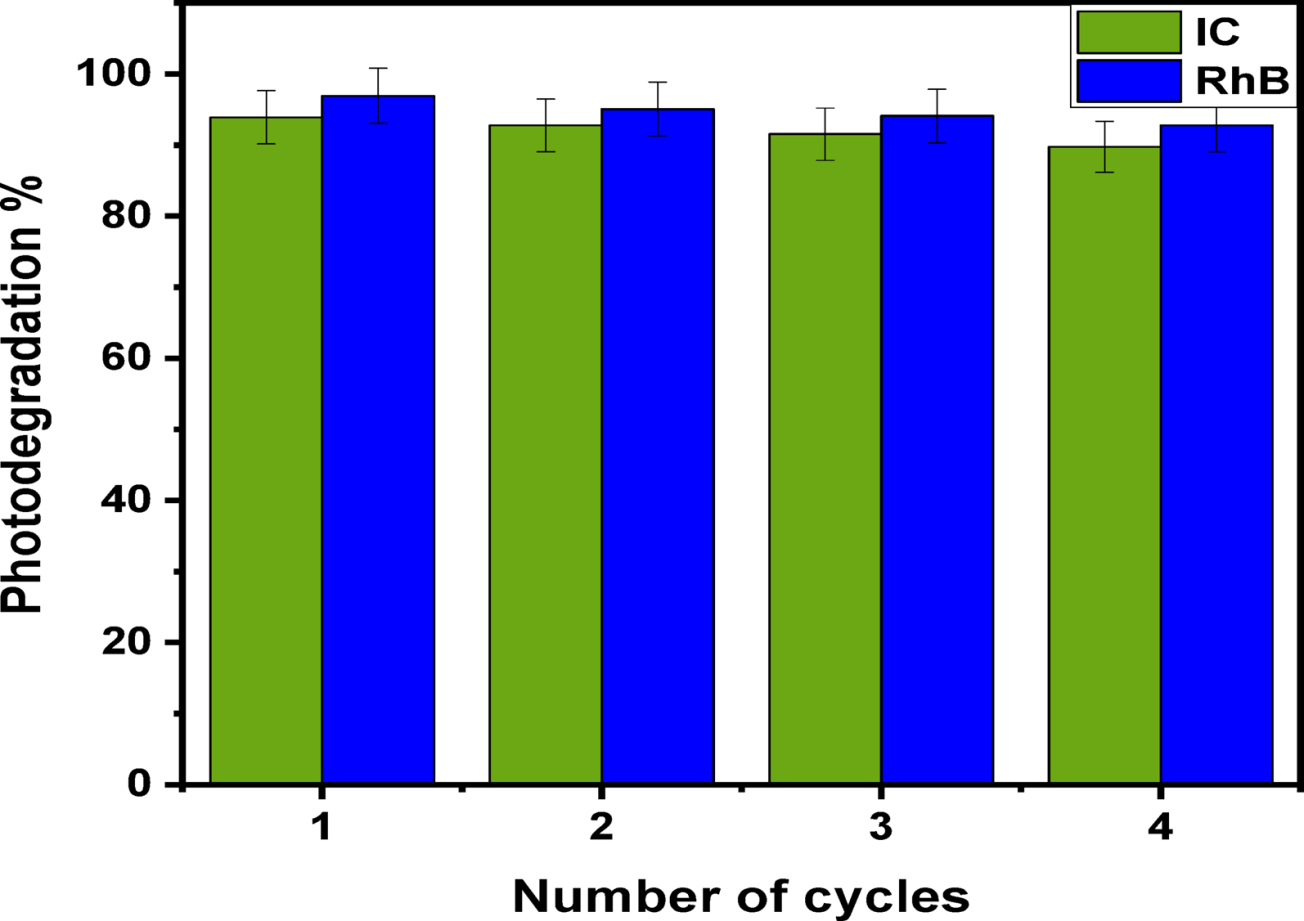
The probability for reusing of (Ag)Kao/ZnO-QDs nanoparticle in dye removal process of IC and RhB dyes

The efficiency of nanostructure once it uses many times is largely dependent on how well the (Ag)Kao/ZnO-QDs nanocomposite performs when it uses in actual large-scale production procedures. The (Ag)Kao/ZnO-QDs nanoparticle’s persistence has been evaluated through three rounds of recycling research of the dye removal process of IC and RhB dyes. prior to being employed again in further dye removal studies, the nanocomposite’s suspension was centrifugated after each test, treated three times using ethanol as well as distilled water and finally dried in an oven set at 80 °C for 12 h. As shown in Fig. 16, the photocatalytic degradation efficacy of IC and RhB dyes was seemed to have slightly declined after the three reprocessing runs of the (Ag)Kao/ZnO-QDs nanostructure compared to the initial ratio from 93.92 to 92.78, 91.54 and 89.76% in addition to the main ratio from 96.93 to 95.08, 94.11 and 92.58%, respectively. This is because the effectiveness of the investigated nanoparticle demonstrated a significant level of constancy during the reuse processes below direct sunlight [77, 78].

### Comparison with current works of literature

As indicated in Table 1, earlier studies carried out under the same testing conditions have been compared with various photocatalysts in order to assess the current dye removal efficacy of IC and RhB dyes utilizing the (Ag)Kao/ZnO-QDs nanomaterial. The (Ag)Kao/ZnO-QDs nanostructure was demonstrated to have superior performance, safe features, greater consistency, cheaper prices and small influence on the climate when compared to all of the previously published composites.

**Table 1:** Assessment the effectiveness of different photocatalysts in dye removal technique compared with the IC and RhB dyes

Composite	Surface area, Band gap	Dye	Catalyst amount	Dye ratios, Irradiation time	Degradation efficacy (%)	Reference
Kao-Ag_2_O/QDs	2.9 eV, 53.3005 m^2^g^− 1^	IC	1500 mg/L	20 mg/L, 90 min (sunlight)	93.46	[43]
TiO_2_ QDs	3.2 eV, 45.791 m^2^g^− 1^	IC	750 mg/L	10 mg/L, 60 min (UV)	95	[79]
TiO_2_ anatase	8 m^2^g^− 1^	IC	1000 mg/L	23 mg/L, 60 min (mercury-vapour lamp “125 W”)	100	[80]
TiO_2_ nanotubes	76 m^2^g^− 1^	IC	1000 mg/L	23 mg/L, 110 min (mercury-vapour lamp “125 W”)	100	[80]
Bi_5_Ti_3_FeO_15_	2.03 eV	IC	60 mg/L	30 mg/L, 240 min (sunlight)	97	[81]
SrZrO_3_	5.3 eV	IC	500 mg/L	30 mg/L, 240 min (UV)	51	[82]
(Ag)Kao/ZnO-QDs	2.89 eV, 50.3418 m^2^g^− 1^	IC	2000 mg/L	30 mg/L, 150 min (sunlight)	93.92	current study
Kao-Ag_2_O/QDs	2.9 eV, 53.3005 m^2^g^− 1^	RhB	2000 mg/L	10 mg/L, 90 min (sunlight)	91.09	[43]
ZnO NPs	3.29 eV, 7.8703 m^2^g^− 1^	RhB	1500 mg/L	10 mg/L, 70 min (UV illumination)	95	[83]
TiO_2_-QDs-Kao	2.85 eV, 107.724 m^2^g^− 1^	RhB	1400 mg/L	5 mg/L, 120 min (sunlight)	91	[42]
TiO_2_ films	50 m^2^g^− 1^	RhB	650 mg/L	20 mg/L, 180 min (UV radiation)	80	[84]
graphitic carbon nitride (g-C_3_N_4_)	2.75 eV, 25.45 m^2^g^− 1^	RhB	1000 mg/L	24 mg/L, 320 min (sunlight)	95	[85]
Pb_3_Nb_4_O_13_/Fumed SiO_2_	2.96 eV, 260.3 m^2^g^− 1^	RhB	3000 mg/L	12 mg/L, 120 min (visible light)	86	[86]
(Ag)Kao/ZnO-QDs	2.89 eV, 50.3418 m^2^g^− 1^	RhB	2500 mg/L	10 mg/L, 150 min (sunlight)	96.93	current study

## Optimization technique

The Box-Behnken design (BBD) was used to maximize each of the primary photodegradation parameters like: pH, time and photocatalyst’s dosage. BBD is a popular technique for designing investigations that uses multiple tests to provide a secure and realistic evaluation of the intended result. Once the most fitting theoretical design has been generated, the dye removal ratios of the IC and RhB dyes in the process are maximized by checking the duration, dose and pH various factors in their optimal ratios (Tables 2 and 3). Using the BBD and Stat-Ease, Design-Expert programs, each examine was put up [87].

**Table 2:** Evaluations, Estimation rates and simulation frameworks for IC dye removal process

Run	Time (min)	pH	Dose (g)	Degradation %
1	95	5	0.3	81.6
2	95	9	0.3	80.2
3	95	7	0.175	89
4	180	7	0.3	87.6
5	95	7	0.175	89
6	95	5	0.05	82.6
7	10	7	0.3	7.3
8	180	5	0.175	92.92
9	10	7	0.05	8.4
10	180	7	0.05	88.1
11	95	7	0.175	89
12	10	9	0.175	10.4
13	10	5	0.175	7.6
14	95	9	0.05	79.4
15	95	7	0.175	89
16	95	7	0.175	89
17	180	9	0.175	91.3

**Table 3:** Evaluations, Estimation rates and simulation frameworks for RhB dye removal process

Run	Time (min)	pH	Dose (g)	Degradation %
1	180	7	0.3	94.92
2	180	7	0.05	86.2
3	95	7	0.175	89.1
4	95	9	0.05	81.6
5	10	7	0.05	2.1
6	95	7	0.175	89.1
7	10	5	0.175	5.4
8	10	9	0.175	7.6
9	180	9	0.175	89.9
10	95	5	0.05	80.7
11	95	9	0.3	90.6
12	95	7	0.175	89.1
13	95	7	0.175	89.1
14	10	7	0.3	7.1
15	180	5	0.175	90.6
16	95	7	0.175	89.1
17	95	5	0.3	91.2

**Fig. 17:**
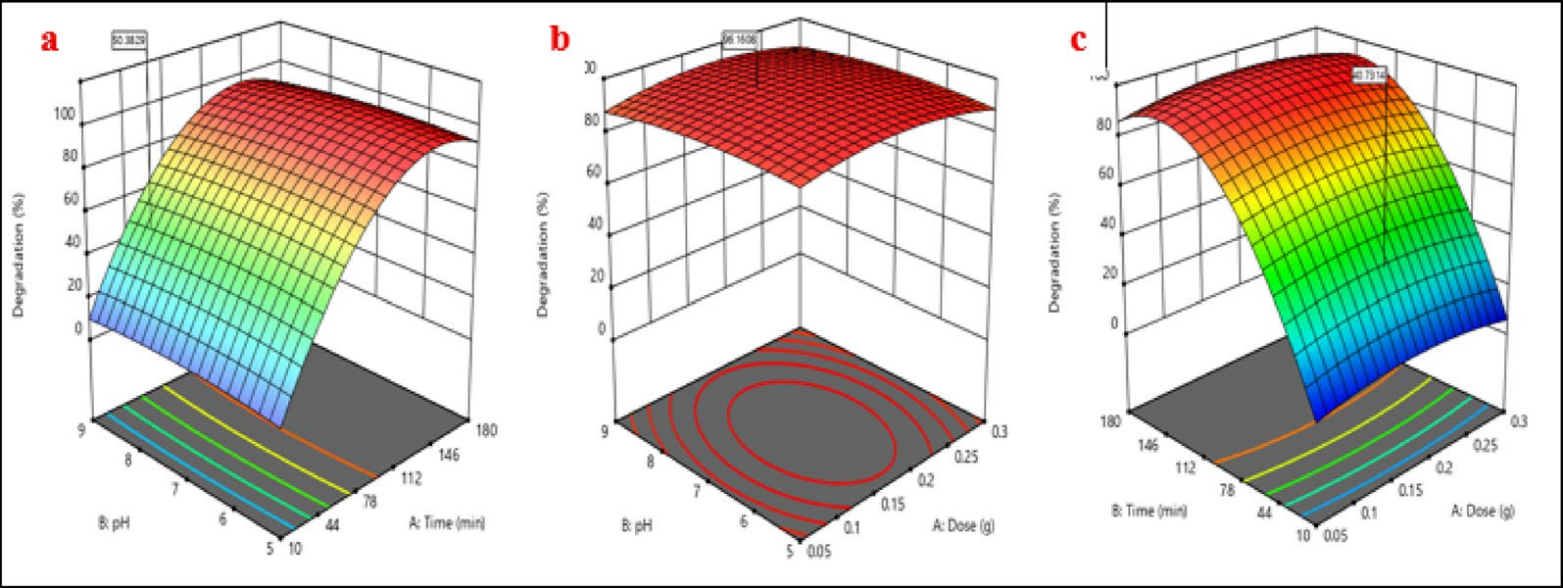
3D responsive surface plots of (a) pH and time (b) photocatalyst’s dosage and pH and (c) photocatalyst’s dosage and time for IC dye removal method utilizing (Ag)Kao/ZnO-QDs nanostructure

To determine significant relationships between every of the parameters under investigation and to understand how the items under investigation affect the photodegradation method of IC and RhB dyes, the three-dimensional (3D) responsive surface plots were created. Figure 17 displays the 3D responsive surface plots for the photocatalytic degradation performance of IC dye utilizing (Ag)Kao/ZnO-QDs nanocomposite. According to the graph, the quantity of the dosage and the time have a major influence upon the research but the pH of the dye solution has a little effect on the photodegradation rate. At a fixed amount of (Ag)Kao/ZnO-QDs photocatalyst, the diagram indicates how pH and time dye affect the efficiency of dye removal process of IC dye as illustrated in Fig. 17(a). The findings showed that rising the period improved the photodegradation efficacy of IC dye with a little effect of pH. Figure 17(b) illustrates how the amount of (Ag)Kao/ZnO-QDs photocatalyst plus pH dye solution affect the photocatalytic degradation rate of IC dye over a predetermined period of time. It was found that the photocatalytic degradation gradually increases when the dose quantity rises to achieve the maximum removal effectiveness at a dosage of 0.2 g after which it slightly decreases. The findings displayed in Fig. 17(c) demonstrate that the effectiveness of IC degradation is significantly influenced by the mass of the (Ag)Kao/ZnO-QDs photocatalyst and the period of time spent at a certain pH level of IC dye. This figure shows the increase in IC dye elimination efficiency over time. Growing the (Ag)Kao/ZnO-QDs nanostructure’s mass from 0.05 to 0.2 g also improved the photocatalytic degradation process of IC dye before declining [88].

**Fig. 18:**
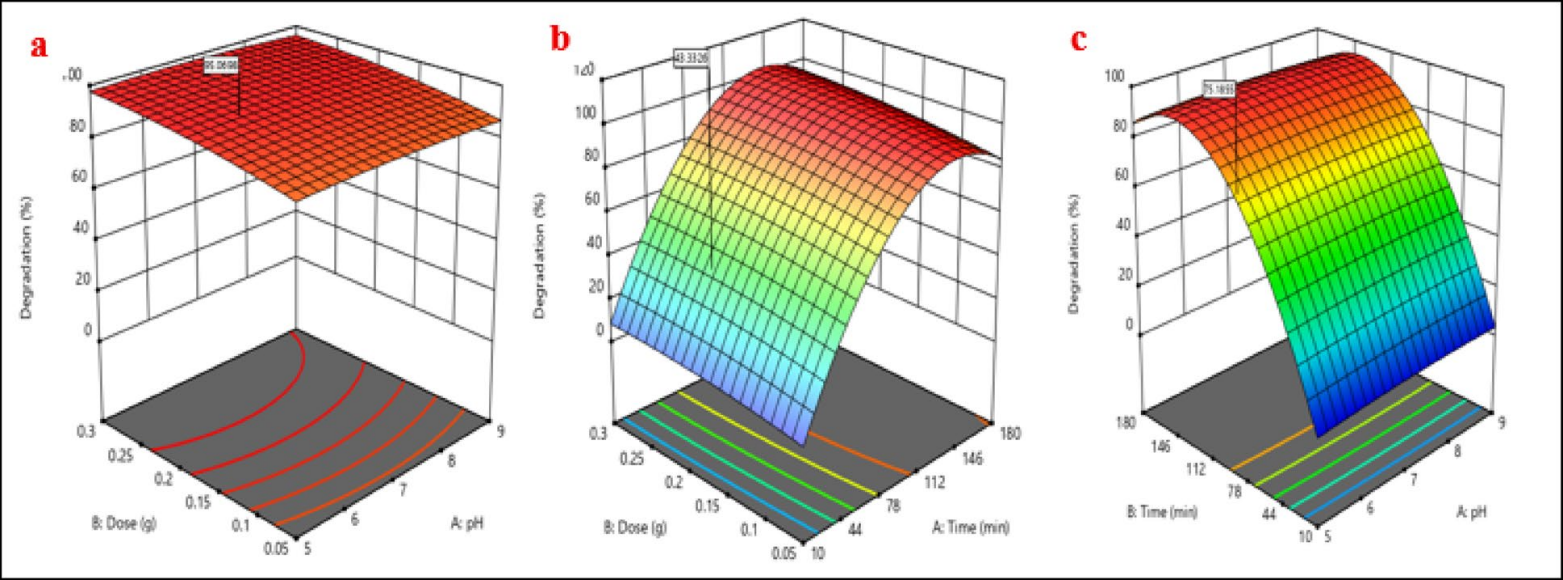
3D responsive surface plots of (a) pH and photocatalyst’s dosage, (b) time and photocatalyst’s dosage and (c) pH and time for RhB dye removal method utilizing (Ag)Kao/ZnO-QDs nanostructure

Figure 18 displays three-dimensional responsive surface plots for the photodegradation efficacy of RhB dye utilizing (Ag)Kao/ZnO-QDs nanocomposite. The diagram indicates that while time and amount of the nanocomposite have a significant impact on the study, the dye solution’s pH has minimal behavior over the photocatalytic degradation performance. The influence of pH colorant solution as well as (Ag)Kao/ZnO-QDs photocatalyst dosage on RhB removal efficiency during a specified time is shown in Fig. 18(a). It was discovered that as the amount of the photocatalyst rises, RhB dye photodegradation progressively growths before marginally reduces. The effect of both time and mass of (Ag)Kao/ZnO-QDs photocatalyst on the RhB dye removal performance is shown in Fig. 18(b). This graphic clearly illustrates how the effectiveness of the RhB pigment photocatalytic degradation rises over time. It is clear that RhB dye decomposition efficacy increased as the nanocomposite concentration increased prior declining. Figure 18(c) illustrates how period as well as pH with a given concentration of (Ag)Kao/ZnO-QDs nanocomposite are related to the efficiency of RhB dye decomposition. It has been shown that at a fixed amount of the photocatalyst, the photocatalytic degradation of RhB dye increases with time with a small effect of pH (Table 3) [89].

## Conclusion

The photodegradation characteristics of the produced (Ag)Kao/ZnO-QDs nanostructure were examined in this research by measuring the efficiency by which rhodamine B (RhB) and indigo carmine (IC) dyes were removed from aqueous solutions below sunlight irradiation. ZnO plus Ag_2_O quantum dots, less than 5 nm, were doped across the nanopores of teeny kaolinite nanosheets using a quick, effective and affordable thermal manufacturing method. A total pore volume of 0.0769 cc/g, an enhanced particular surface area of 50.3148 m^2^g^− 1^and a pore size of 3.0565 nm were all characteristics of the generated nanostructure. Furthermore, 2.89 eV was found to be the optical band gap energy. The (Ag)Kao/ZnO-QDs nanostructure’s point of zero charge (pzc) was found at pH = 5.3. Following 150 min of exposition to solar irradiation, the study indicates that the (Ag)Kao/ZnO-QDs nanostructure with optimal dose of 2000 and 2500 mg/L achieved the highest removal performance IC and RhB dyes at 93.92 and 96.93%, respectively, at neutral conditions (pH = 7). The dye removal process of IC and RhB dyes followed first-order kinetics with rate constants of 0.0166 and 0.018 min^− 1^ at pH = 7, individually. Scavenger radical tests have demonstrated that superoxide radicals (^•^O_2_^−^) are essential to the mechanism of the decomposition of IC dye, while superoxide radicals (^•^O_2_^−^) plus hydroxyl radicals (^•^OH) are necessary for the photocatalytic degradation of RhB dye. After triple reuse studies, it was showed that the (Ag)Kao/ZnO-QDs nanoparticle was highly stable which evidenced by the photodegradation efficacy of IC and RhB dyes which went from 93.92 to 92.78, 91.54, and 89.76% as well as from 96.93 to 95.08, 94.11 and 92.58%, individually. The Box-Behnken design (BBD) which identified the optimal values for a number of variables was appropriate for the photocatalytic degradation tests.

## Supplementary Information

Below is the link to the electronic supplementary material.

Supplementary Material 1

## Data Availability

The data that support the findings of this study are available from the corresponding author upon reasonable request.
