# Supplementary material for: Enhancement of commercial dye photocatalytic degradation through the use of silver-doped kaolinite-zinc oxide quantum dots
Source: BMC Chem. 2025 Oct 29;19(1):291. doi: 10.1186/s13065-025-01648-2 (PMC12574229; doi:10.1186/s13065-025-01648-2)
Supplement: Supplementary file 1 — Supplementary Material 1 [file 13065_2025_1648_MOESM1_ESM.docx]

Fig. S1. shows the correlation between the concentration (ppm) with absorption (nm) readings of the IC dye throughout calibration.

Fig. S2. shows the correlation between the concentration (ppm) with absorption (nm) readings of the RhB dye throughout calibration.

List of abbreviations.

| Abbreviation | Meaning |
| --- | --- |
| RhB | Rhodamine B dye |
| IC | Indigo carmine dye |
| (Ag)Kao/ZnO-QDs | silver-doped kaolinite-zinc oxide quantum dots nanocomposite |
| ^•^O_2_^−^ | superoxide radicals |
| ^•^OH | hydroxyl radicals |
| WHO | World Health Organization |
| Kao | Kaolinite clay |
| h^+^ | Holes |
| e^−^ | Electrons |
| UV | Ultraviolet |
| µm | Micrometer |
| nm | Nanometer |
| °C | Celsius degree |
| g | Gram |
| XRD | X-ray diffraction |
| eV | Electron volt |
| XPS | X-ray photoelectron spectroscopy |
| FTIR | F[ourier transform infrared](https://www.sciencedirect.com/science/article/pii/B9780444637765000012) |
| SEM | Scanning electron microscope |
| TEM | Transmission electron microscope |
| SAED | Selective area electron diffraction |
| kV | Kilo volt |
| EDX | Energy-dispersive X-ray spectroscopy |
| DLS | Dynamic Light Scattering |
| FPS | Frequency Power Spectrum |
| M | Molar |
| BET | Brunauer-Emmett-Teller |
| BJH | Barrett-Joyner-Halenda |
| DRS | diffuse reflectance spectroscopy |
| mg | Milligram |
| L | Liter |
| mL | Milliliter |
| A_0_ | Initial absorbance |
| A_t_ | Final absorbance |
| C_0_ | Initial concentration |
| C_t_ | Final concentration |
| T | Time |
| A | Absorbance |
| C | Concentration |
| Eq | Equation |
| S | Supplemental |
| D% | Photocatalytic degradation rate |
| K | Photodegradation rate constant |
| min | Minutes |
| Fig | Figure |
| D_001_ | crystallite size |
| HI | crystallinity index |
| Pzc | point of zero charge |
| mV | Millivolt |
| E_g_ | band gap energy |
| λ_g_ | absorbance wavelength |
| R^2^ | correlation coefficients |
| VB | Valence band |
| CB | Conductive band |
| BBD | Box-Behnken Design |
| 3D | Three-dimensional responsive surface plot |
